# Supplementary material for: KANK1 promotes breast cancer development by compromising Scribble-mediated Hippo activation
Source: Nat Commun. 2024 Nov 29;15:10381. doi: 10.1038/s41467-024-54645-9 (PMC11607453; doi:10.1038/s41467-024-54645-9)
Supplement: Supplementary file 1 — Supplementary Information [file 41467_2024_54645_MOESM1_ESM.pdf]

Supplementary Information

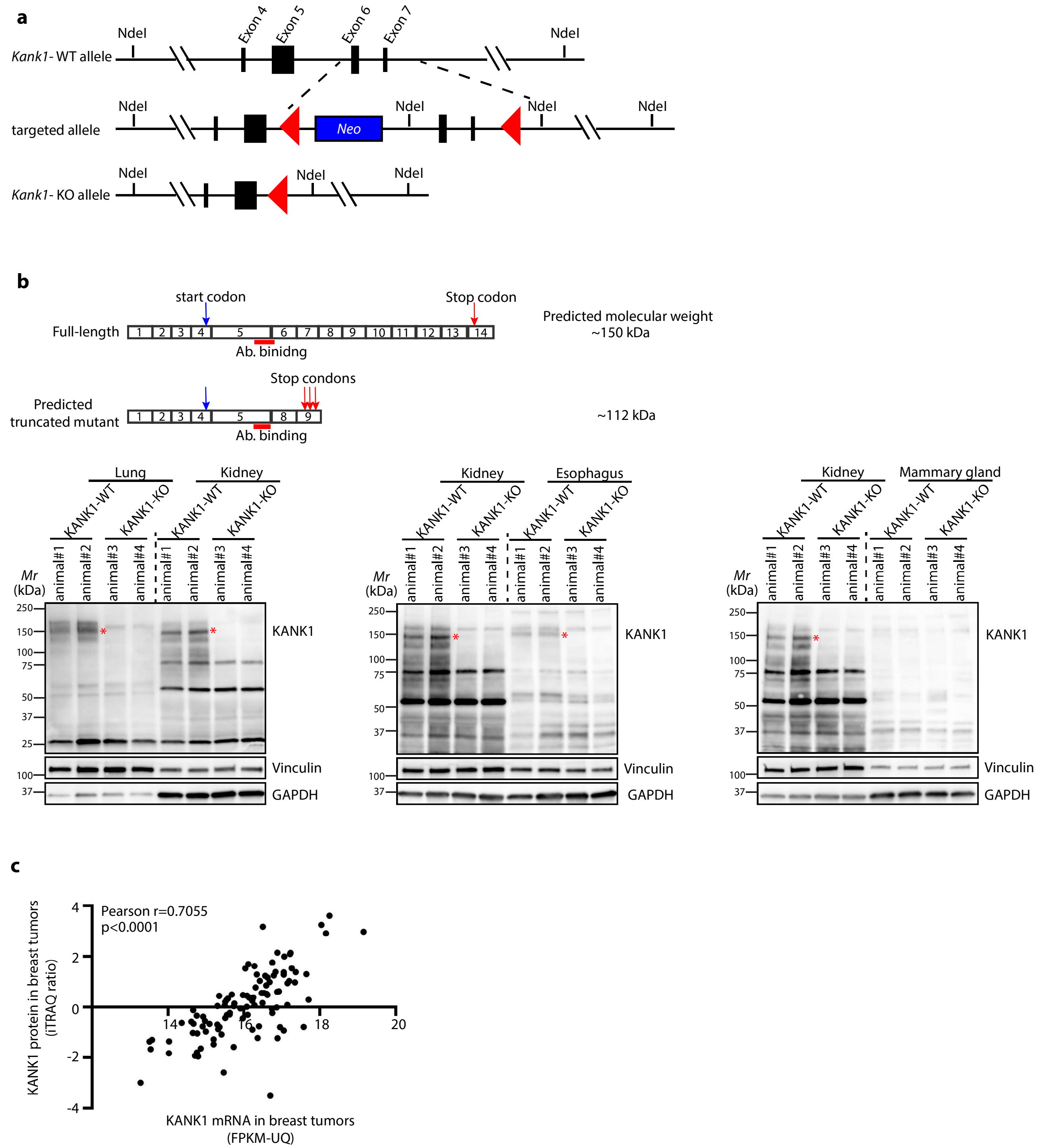

Supplementary Figure 1: Generation of KANK1-KO mouse strain

(a) Scheme depicting the targeting strategy to obtain the Kank1-KO allele. **(b)** Scheme depicting the exon composition of full-length and predicted truncated mutant of KANK1. Start codon localizes at exon 4 and stop codon at exon 14 in full-length KANK1. Cre-mediated deletion of exon 6 and 7 resulted in multiple stop codons generated at exon 9. Tissue lysates from lung, kidney, esophagus and mammary gland from two litter mates were analyzed by WB. KANK1 protein and the possible truncated mutant of KANK1 were examined with a commercial antibody recognizing exon 5 of KANK1 (indicated in red). **(c)** Correlation between KANK1 mRNA level and protein level from 105 breast tumors. mRNA level expressed as FPKM-UQ (Fragments Per Kilobase per Million mapped reads-Upper Quartile) and extracted from TCGA-BRCA.htseq_fpkm-uq.tsv, protein level expressed as iTRAQ ratio (Isobaric tag for relative and absolute quantitation) and extracted from previous publication70. Pearson correlation coefficient (r) analysis and *P* value (*t*-distribution with n-2 degrees of freedom) calculation were performed in GraphPad Prism.
Source Data are provided as a Source Data file.

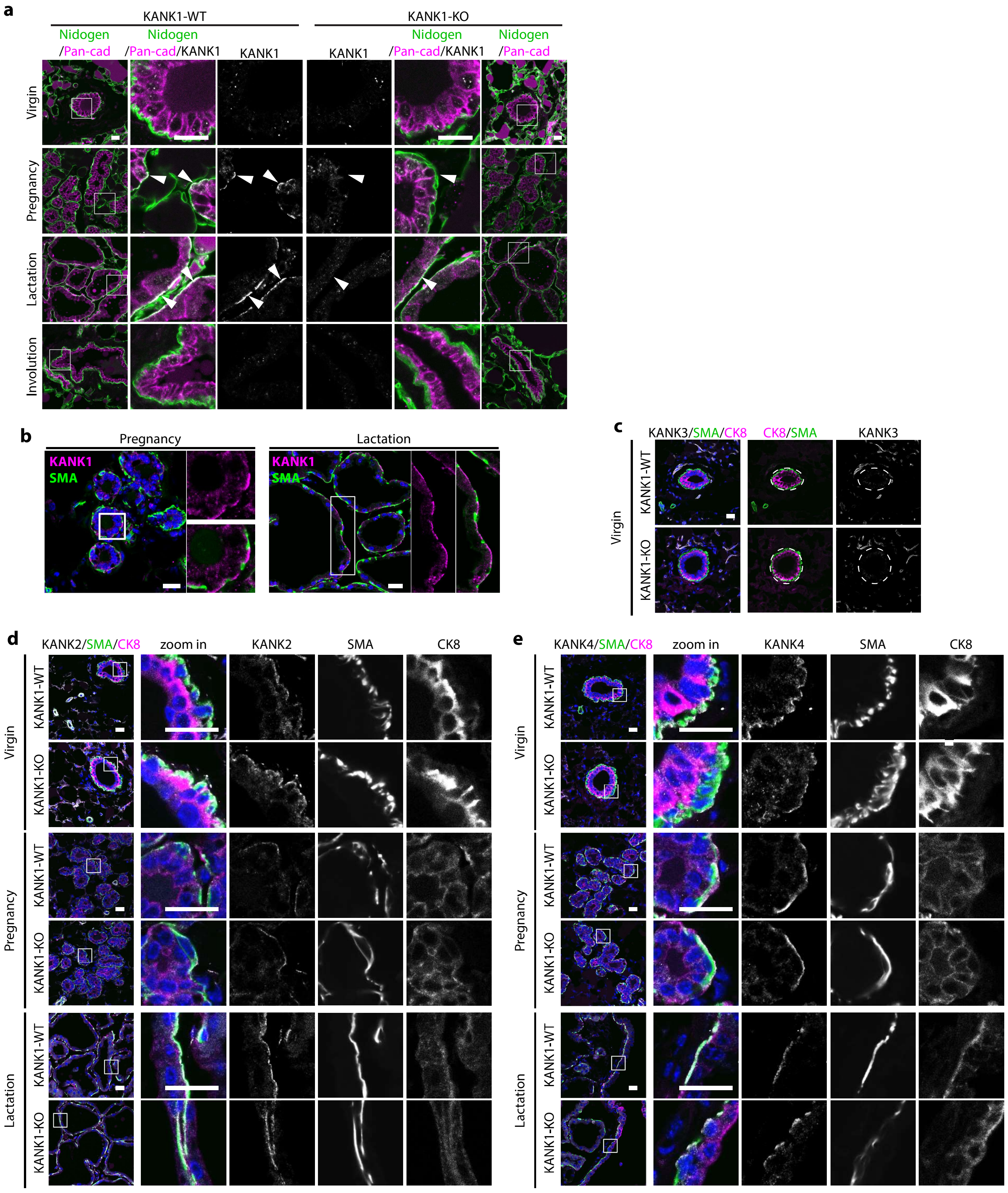

Supplementary Figure 2: KANKs expression in the mouse mammary gland

(a) Immunostaining of KANK1-WT and KANK1-KO MG sections at indicated life phase for KANK1 (white), Nidogen (green, BM marker) and Pan-cadherin (Pan-cad, magenta, epithelial cell marker). The basal sides with positive KANK1 immunosignals are indicated with white arrow heads. Scale bars: 20 µm. **(b)** Immunostaining of KANK1-WT MG sections from pregnant and lactating mice for KANK1 (magenta) and SMA (green, myoepithelial cell marker). Nuclei were counterstained with DAPI (blue). Scale bars: 20 µm. **(c-e)** Immunostaining of KANK1-WT and KANK1-KO MG sections at indicated life phase for KANK3 (**c**), KANK2 (**d**), KANK4 (**e**) (white), SMA (green) and cytokeratine 8 (CK8, magenta, luminal epithelial cell marker). Nuclei were counterstained with DAPI (blue). Scale bars: 20 µm.

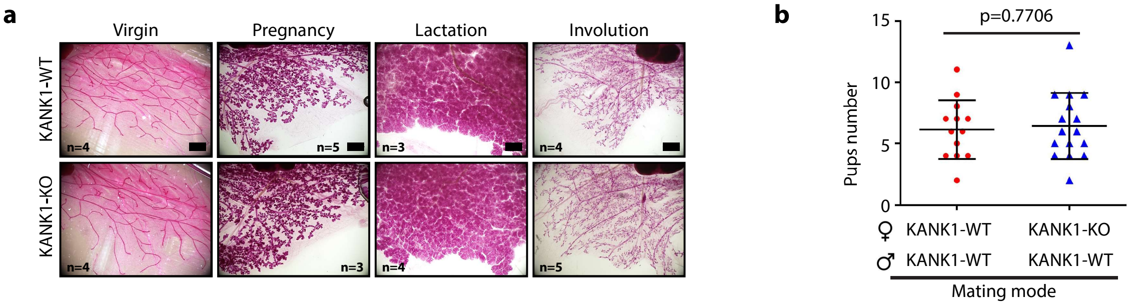

Supplementary Figure 3: Mammary gland morphology in KANK-WT and KANK1-KO females

(a) Representative whole mount Carmine Alum staining of KANK1-WT and KANK1-KO MG at indicated life phase. Number of animals stained and analyzed is indicated in figures. Scale bars: 1 mm. **(b)** Pup numbers at weaning stage from KANK1-WT and KANK1-KO females mated with KANK1-WT males. *P* values were calculated using unpaired Student's *t* test. Mean ± SD is shown.

Source Data are provided as a Source Data file.

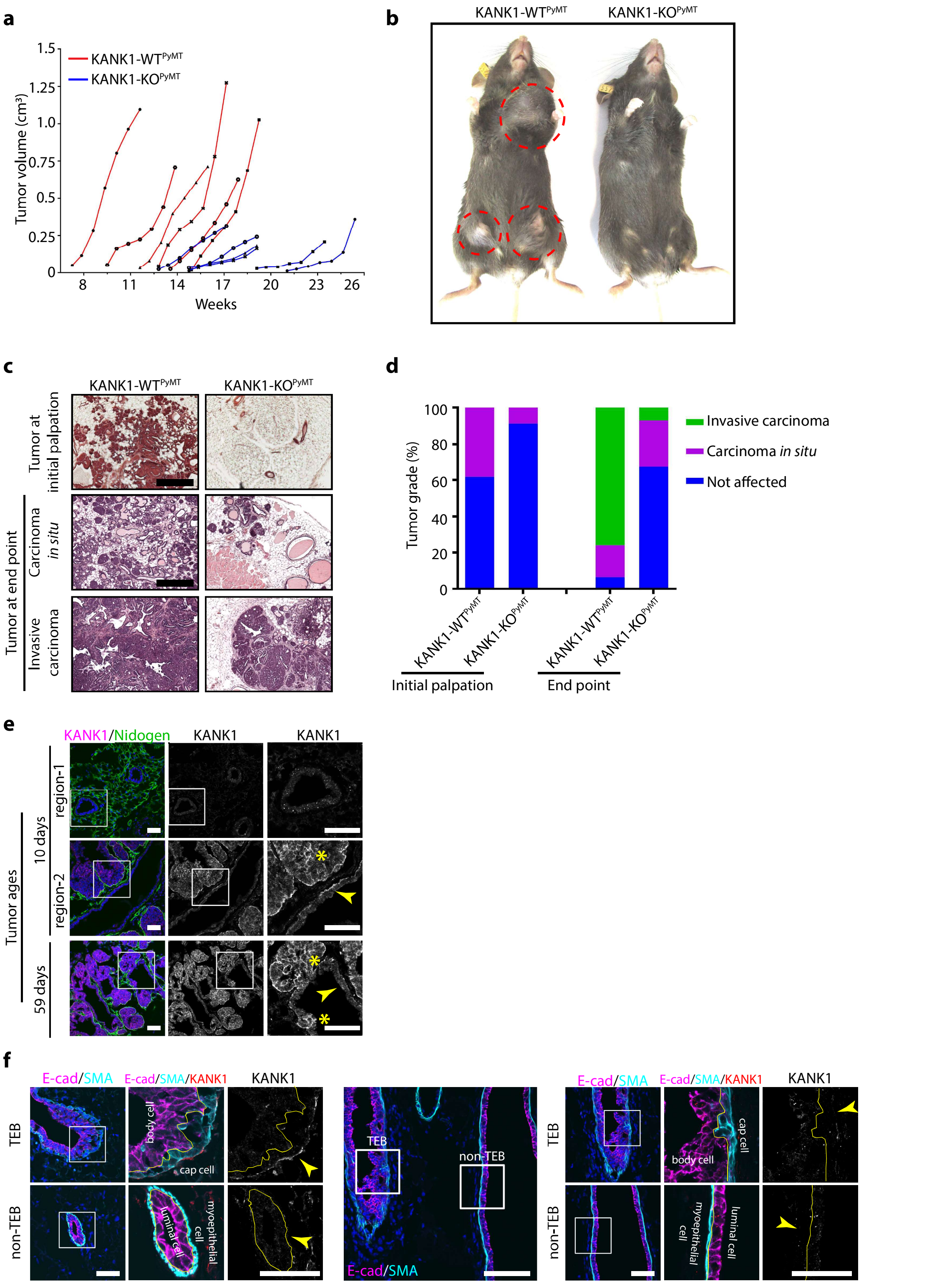

Supplementary Figure 4: Tumor development in KANK1-WT^{PyMT} and KANK1-KO^{PyMT} mice
(a) Tumor growth curve in KANK1-WT^{PyMT} (red) and KANK1-KO^{PyMT} (blue) mice (n=6 litter mates) (see also Fig. 2c for growth rate quantification). **(b)** Representative image of tumors from littermate mice at the experimental end point of 16 weeks after first palpation. The visible KANK1-WT^{PyMT} tumors are indicated with red circles. The tumors in KANK1-KO^{PyMT} mice are palpable, small and not visible. **(c,d)** Hematoxylin-eosin H/E staining (**c**) and grading (**d**) of tumors developed in KANK1-WT^{PyMT} and KANK1-KO^{PyMT} littermates. Analysis was performed at day 10 after initial palpation of tumors (n=3 for KANK1-WT^{PyMT} and n=4 for KANK1-KO^{PyMT}) and at the experimental end point of 16 weeks (n=9 for KANK1-WT^{PyMT} and n=8 for KANK1-KO^{PyMT}). Scale bars: 0.5 mm. **(e)** Immunostaining of KANK1-WT^{PyMT} tumor sections at the tumor stage of either 10 days (animal age: 11.7 weeks) or 59 days (animal age: 16 weeks) after palpation for KANK1 (magenta) and Nidogen (green). KANK1 localizes at basal side (arrow heads) and cell-cell junctions (asterisks) of tumor cells. Nuclei were counterstained with DAPI (blue). Scale bars: 50 µm. **(f)** Immunostaining of KANK1-WT MG isolated from 5-weeks old females for KANK1 (red), SMA (to co-stain cap cells and myoepithelial cells; cyan) and E-cad (to co-stain body cells and luminal cells; magenta). KANK1 immunosignals are only observed in cap cells localized at TEB front and not in other cells. Yellow lines outline the boundary between cap and body cells or between luminal and myoepithelial cells. Nuclei were counterstained with DAPI (blue). TEB: terminal end bud. Scale bars: 50 µm.
Source Data are provided as a Source Data file.

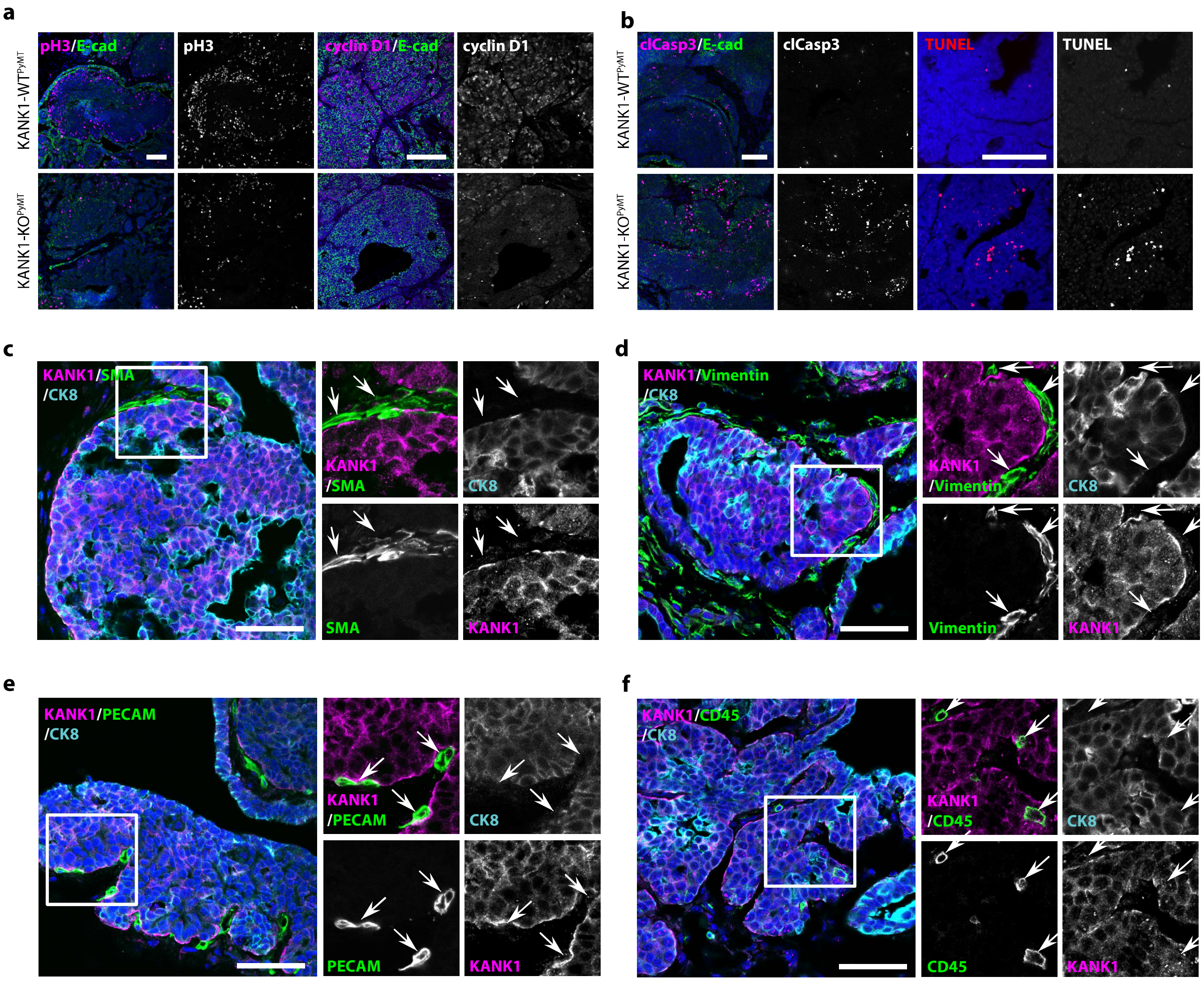

Supplementary Figure 5: Cell proliferation and survival in tumors from KANK1-WT^{PyMT} and KANK1-KO^{PyMT} mice
(a) Cell proliferation analyzed by pH3 and cyclin D1 (magenta) in E-cad+ (green) tumor cells from KANK1-WT^{PyMT} and KANK1-KO^{PyMT} mice (n=5 pairs of littermates). Nuclei were counterstained with DAPI (blue). Quantification is shown in Fig. 2e. Scale bars: 100 µm. **(b)** Cell apoptosis indicated by cleaved Caspase 3 (clCasp3, magenta) in E-cad+ (green) tumor cells and TUNEL (red) analyzed in KANK1-WT^{PyMT} and KANK1-KO^{PyMT} tumor tissues (n=5 pairs of littermates). Nuclei were counterstained with DAPI (blue). Quantification is shown in Fig. 2f. Scale bars: 100 µm. **(c-f)** Immunostaining of KANK1 (magenta), CK8 (cyan) and tumor stroma cell markers SMA (green) for myoepithelial cells and activated fibroblasts (**c**), Vimentin (green) for fibroblasts (**d**), PECAM (green) for endothelial cells (**e**) and CD45 (green) for immune cells (**f**). White arrows indicate regions marking antibody-stained cells that lack KANK1 expression. Nuclei were counterstained with DAPI (blue). Scale bars: 50 µm.

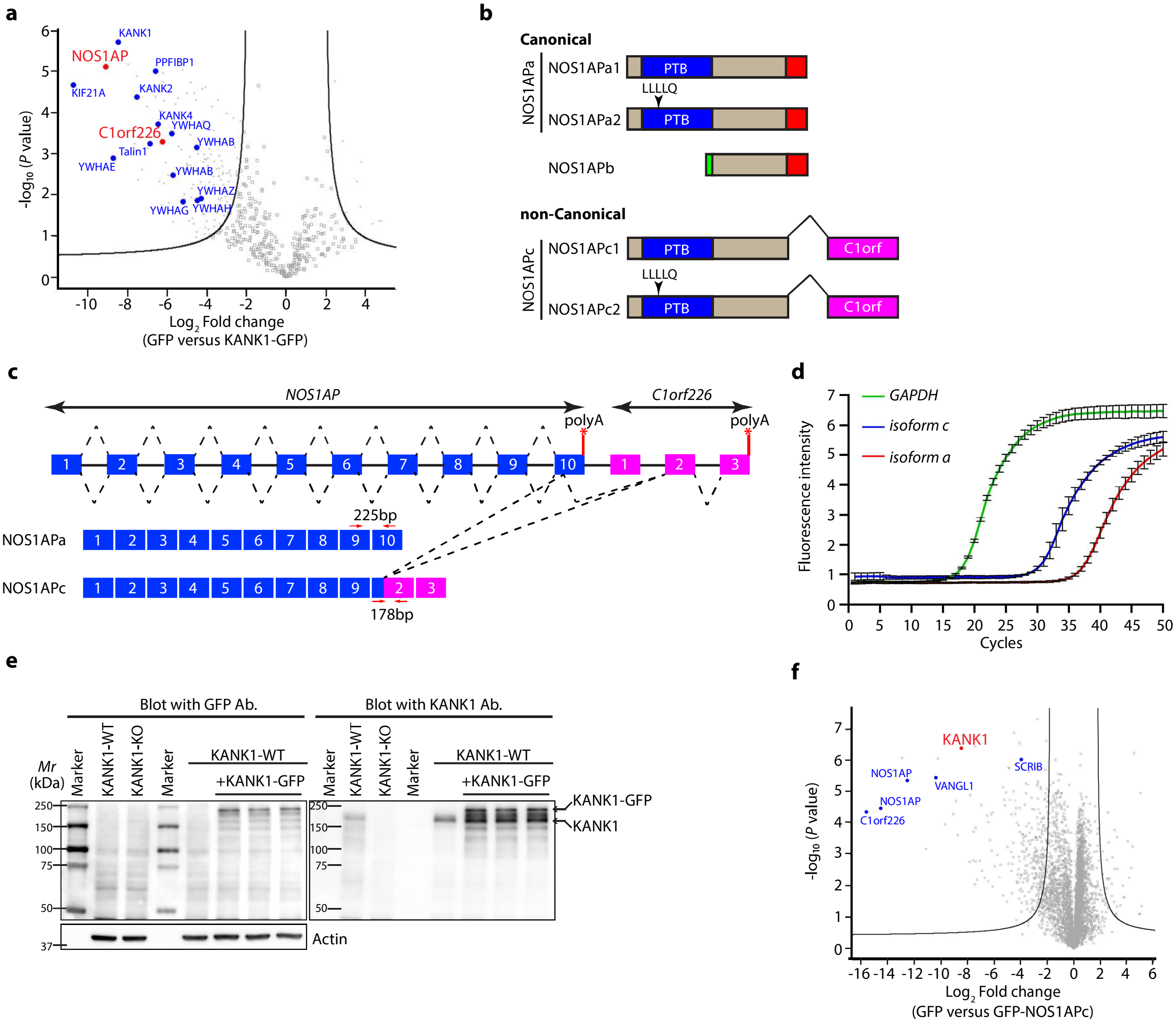

Supplementary Figure 6: Characterization of NOS1AP isoforms
(a) Volcano plot of KANK1-GFP interactors in HEK293T cells (n=3 biological replicates). Known interactors are highlighted in blue, novel interactors in red. *P* values were calculated by two-sided permuted t-test with 250 randomizations. The black line indicates the significance cut-off (FDR:0.01, s0:2) estimated by the Perseus software. **(b)** Cartoon of reported NOS1AP splicing isoforms with PTB domain in blue, NOS1 binding site in red, the small domain specific for NOS1APb in green and the C1orf226 open reading frame (C1orf)-encoded region in magenta. **(c)** Illustration of NOS1AP gene structures and the alternative splice isoforms NOS1APa and NOS1APc. Primers used for qRT-PCR and the corresponding length of the amplicons are indicated with red arrows. **(d)** Amplification curves of qRT-PCR showing relative expression levels of NOS1APa and NOS1APc isoforms in MCF7 cells. GAPDH served as internal control. **(e)** KANK1-GFP identity was confirmed by blotting the membrane with KANK1 antibody after stripping the GFP blotted membrane. KANK1-WT and KANK-KO lysates served as antibody control; Actin served as loading control. **(f)** Volcano plot of GFP-NOS1APc interactors in MCF7 cells (n= 3 biological replicates). Known interactors are highlighted in blue, the novel interactor KANK1 in red. *P* values were calculated by two-sided permuted t-test with 250 randomizations. The black line indicates the significance cut-off (FDR:0.01, s0:2) estimated by the Perseus software.
Source Data are provided as a Source Data file.

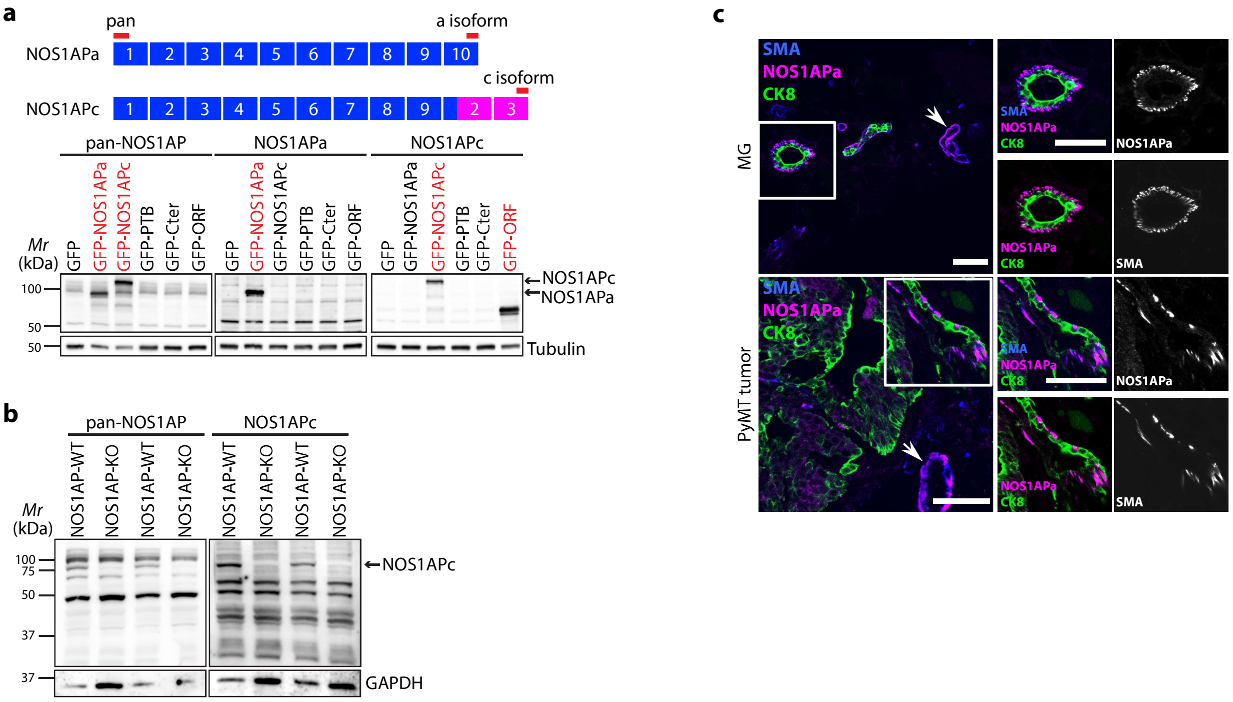

Supplementary Figure 7: Generation of NOS1AP isoform-specific antibodies and expression of NOS1APa at the tissue level
(a) Cartoon location of peptides used for in house antibody generation. WB of lysates from cells expressing indicated GFP-tagged NOS1AP polypeptides with indicated antibodies. Corresponding bands for NOS1APa and NOS1APc were indicated by arrows. Tubulin served as loading control. **(b)** The endogenous expression levels of NOS1AP were examined by immunoblotting of lysates from MCF7$^{NOS1AP\text{-}WT}$ and MCF7$^{NOS1AP\text{-}KO}$ cells with home-made pan-NOS1AP and NOS1APc-specific antibodies. GAPDH served as loading control. **(c)** Immunostaining of KANK1-WT MG and tumor tissues with NOS1APa specific antibody (magenta), SMA (blue) and CK8 (green). Blood vessels are indicated by arrows. Scale bars: 50 µm.

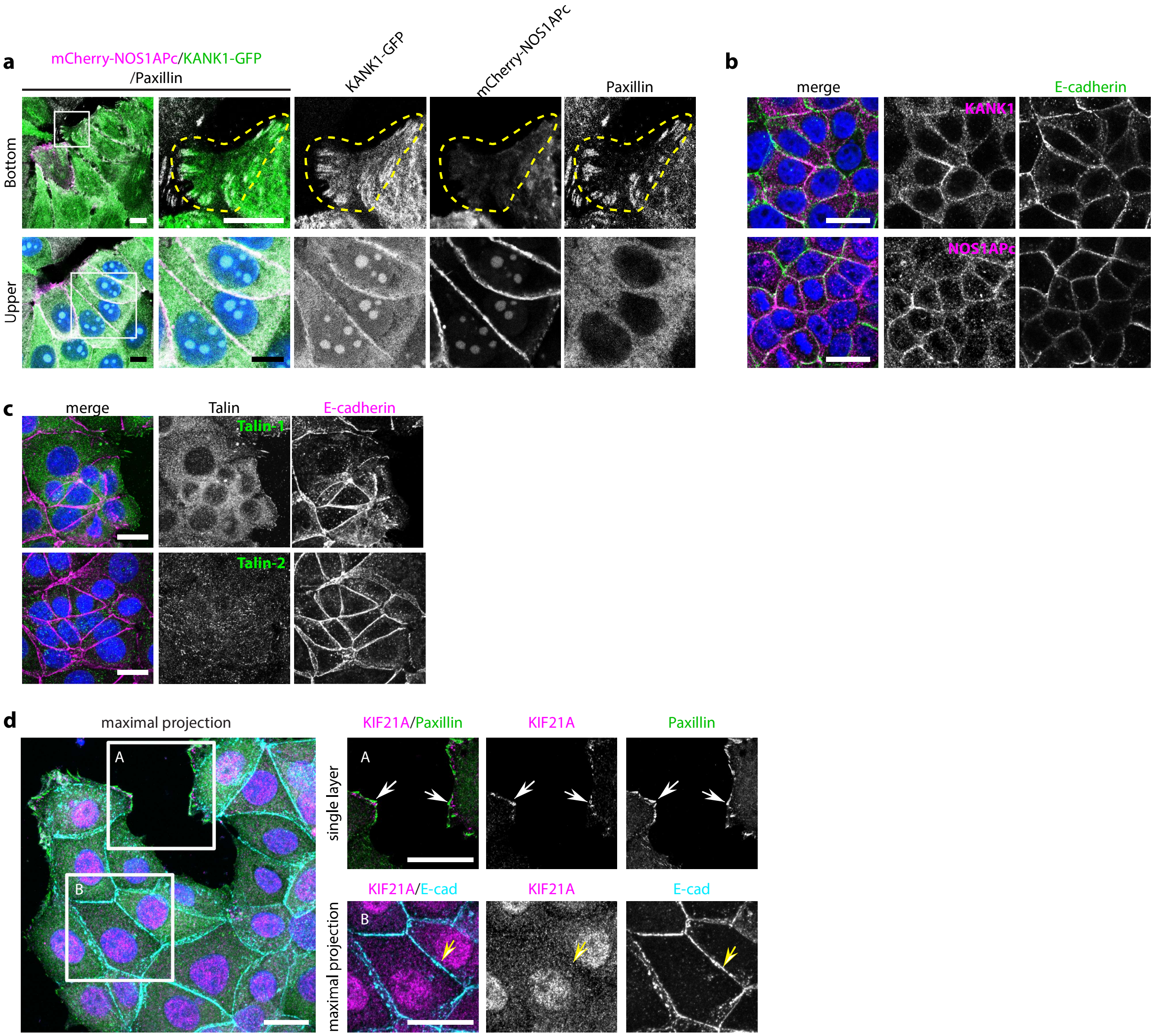

Supplementary Figure 8: Subcellular recruitment of KANK1

(a) MCF7NOS1AP-KO cells reconstituted with mCherry-NOS1APc (magenta) and DOX-inducible KANK1-GFP (green) stained for Paxillin. FAs shown at confocal bottom layer and cell-cell junctions at confocal upper layer. Region of interest in bottom layer is highlighted with yellow dotted lines. Nuclei were counterstained with DAPI (blue). Scale bars: 10 µm. **(b)** Immunostaining of MCF7 cells for KANK1 (magenta), NOS1APc (magenta) and E-cadherin (green). Nuclei were counterstained with DAPI (blue). Scale bars: 20 µm. **(c)** Immunostaining of MCF7 cells for Talin-1 (green), Talin-2 (green) and E-cadherin (magenta). Maximal projection of the signals is shown. Nuclei were counterstained with DAPI (blue). Scale bars: 20 µm. **(d)** IFC staining of MCF7 cells for KIF21A (magenta), Paxillin (green) and E-cadherin (E-cad, magenta). Focal adhesion indicated by white arrows and cell-cell contacts indicated by yellow arrows. Nuclei were counterstained with DAPI (blue). Scale bars: 20 µm.

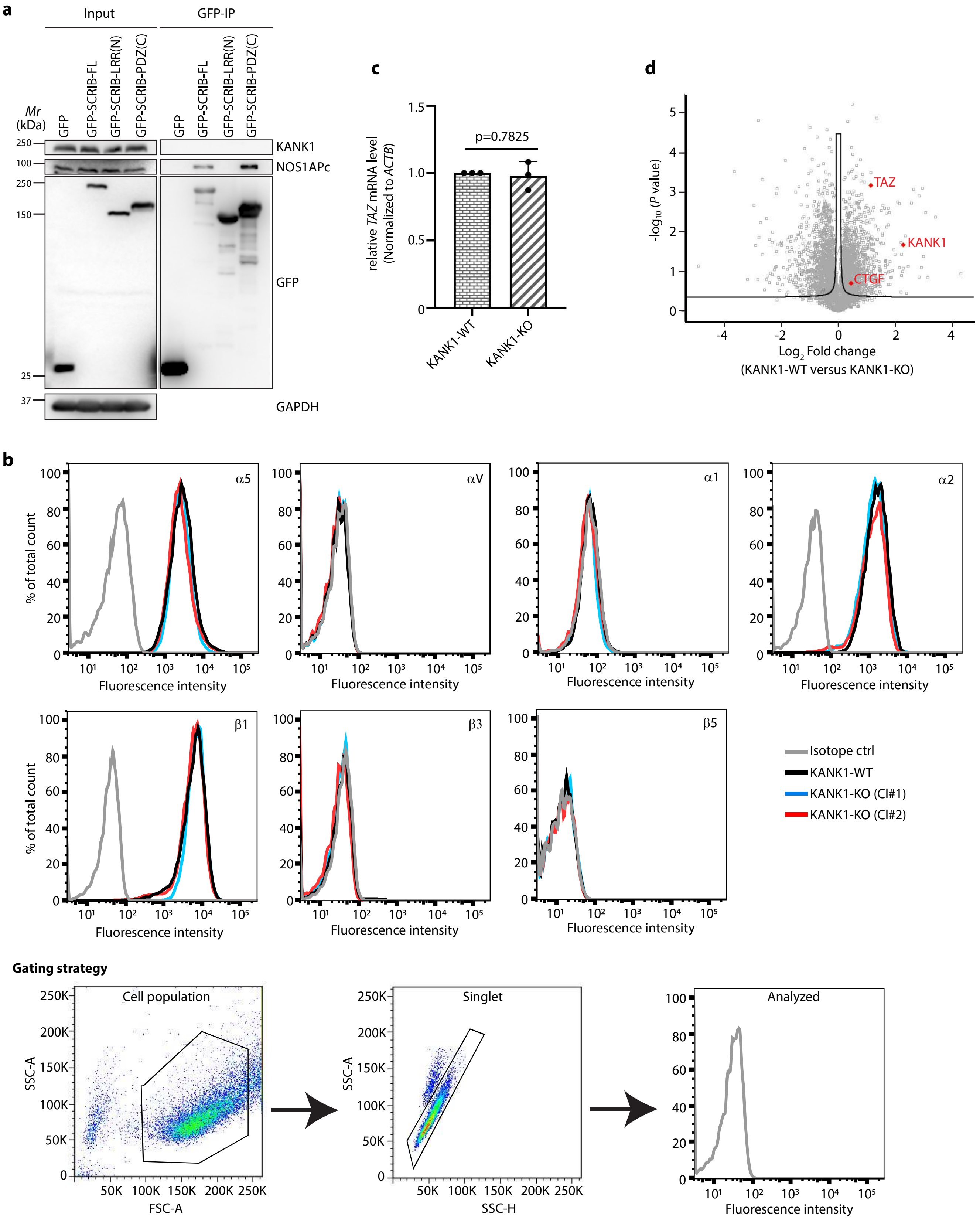

Supplementary Figure 9: Constructs, protein and mRNA expression patterns in $MCF7^{KANK1\text{-}WT}$ and $MCF7^{KANK1\text{-}KO}$ cells

(a) Representative IP of GFP-SCRIB or SCRIB truncation mutants and blotted for KANK1, pan-NOS1AP and GFP. GAPDH served as loading control (n=3 independent experiments). **(b)** Integrin expression profile in $MCF7^{KANK1\text{-}WT}$ and $MCF7^{KANK1\text{-}KO}$ cells. Two independent KANK1-KO clones were analyzed. Indicated integrin subunits were investigated. The same gating strategy was applied for all markers analyed. **(c)** TAZ mRNA expression analyzed by qRT-PCR in $MCF7^{KANK1\text{-}WT}$ and $MCF7^{KANK1\text{-}KO}$ cells after overnight serum starvation. *ACTB* served as internal control (n=3 biological replicates). *P* values were calculated using unpaired Student's t test. Mean ± SD is shown. **(d)** Volcano plot of the total proteome of $MCF7^{KANK1\text{-}WT}$ and $MCF7^{KANK1\text{-}KO}$ cell lysates. Cells were serum starved overnight and then treated with serum for 24 hours before lysis (n=3 biological replicates). *P* values were calculated by two-sided permuted *t*-test with 250 randomizations. The black line indicates the significance cut-off (FDR:0.6, s0:0.08) estimated by the Perseus software.
Source Data are provided in Data Availability section.

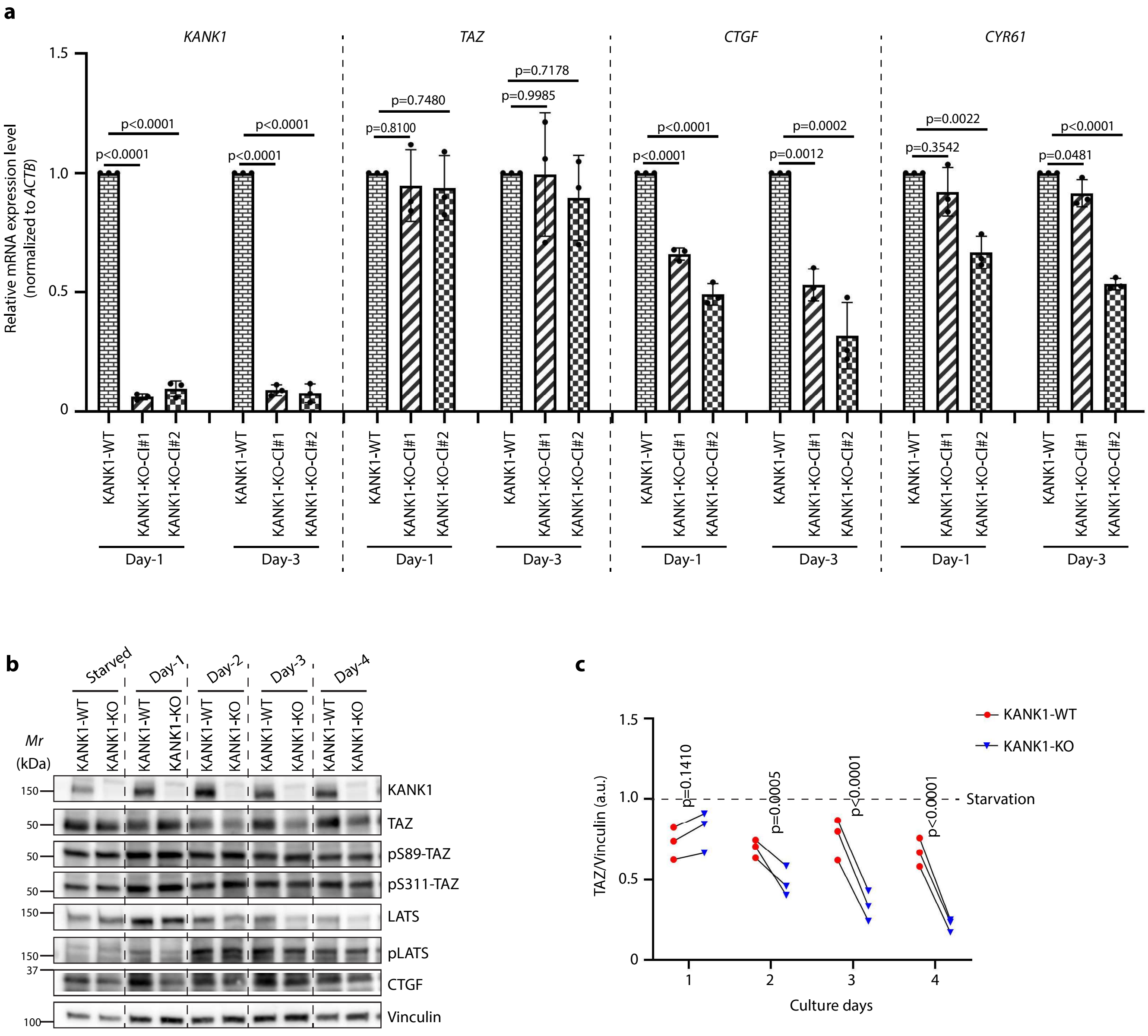

Supplementary Figure 10: Protein and mRNA expression of TAZ and TAZ-targets in MCF7$^{KANK1\text{-}WT}$ and MCF7$^{KANK1\text{-}KO}$ cells
(a) mRNA expression of *KANK1, TAZ, CTGF* and *CYR61* analyzed by qRT-PCR in MCF7$^{KANK1\text{-}WT}$ and MCF7$^{KANK1\text{-}KO}$ cells at day-1 and day-3 after overnight serum starvation. *ACTB* served as the internal control (n=3 biological replicates). *P* values (comparisons to KANK1-WT) were calculated using one-way ANOVA with Dunnett's multiple comparisons test. Mean ± SD is shown. **(b,c)** WB analysis of indicated proteins in lysates of serum-treated MCF7$^{KANK1\text{-}WT}$ and MCF7$^{KANK1\text{-}KO}$ cells after overnight serum starvation. Representative blot from 3 independent experiments. Vinculin served as loading control (**b**). Relative TAZ protein level (normalized to vinculin) is shown in (**c**). TAZ level before serum treatment (Starvation) was set as 1 (a.u.: arbitrary unit). *P* values were calculated using RM two-way ANOVA with Šídák's multiple comparisons test and are indicated on top of comparison.
Source Data are provided as a Source Data file.

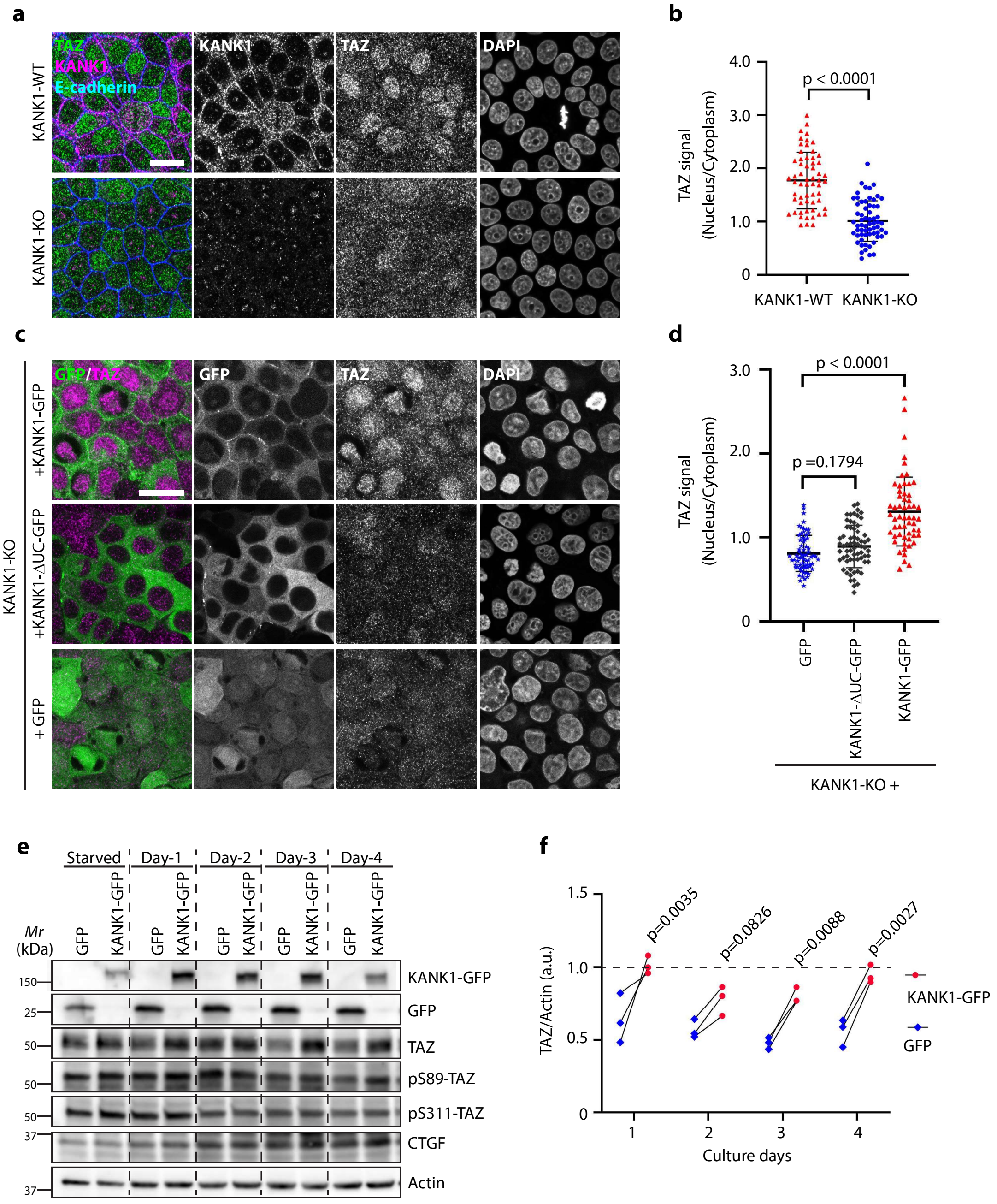

Supplementary Figure 11: KANK1-GFP re-expression restores TAZ stability in $MCF7^{KANK1\text{-}KO}$ cells

(a,b) Immunofluorescence of TAZ (green), KANK1 (magenta) and E-cadherin (blue) in $MCF7^{KANK1\text{-}WT}$ (n=59) and $MCF7^{KANK1\text{-}KO}$ (n=63) cells. Panel (**b**) shows quantification of nuclear TAZ signal intensities. Nuclei were counterstained with DAPI. *P* value was calculated using unpaired Student's t test. Mean ± SD is shown. Scale bar: 20 µm. **(c,d)** Immunofluorescence of TAZ (magenta) in $MCF7^{KANK1\text{-}KO}$ cells reconstituted with KANK1-GFP (green, n=57), KANK1-ΔUC-GFP (green, n=66) or GFP only (green, n=68). Panel (d) shows quantification of nuclear TAZ signal intensities. Nuclei were counterstained with DAPI. *P* value was calculated using one-way ANOVA with Dunnett's multiple comparisons test. Mean ± SD is shown. Scale bar: 20 µm. **(e,f)** WB analysis of lysates from $MCF7^{KANK1\text{-}KO}$ cells reconstituted with KANK1-GFP for indicated times. Relative abundance of TAZ protein levels (normalized to Actin) was quantified in (**f**). TAZ level before serum treatment (Starvation) was set as 1 (a.u.: arbitrary unit). *P* values were calculated using RM two-way ANOVA with Šídák's multiple comparisons test and are indicated on top of each comparison.

Source Data are provided as a Source Data file.

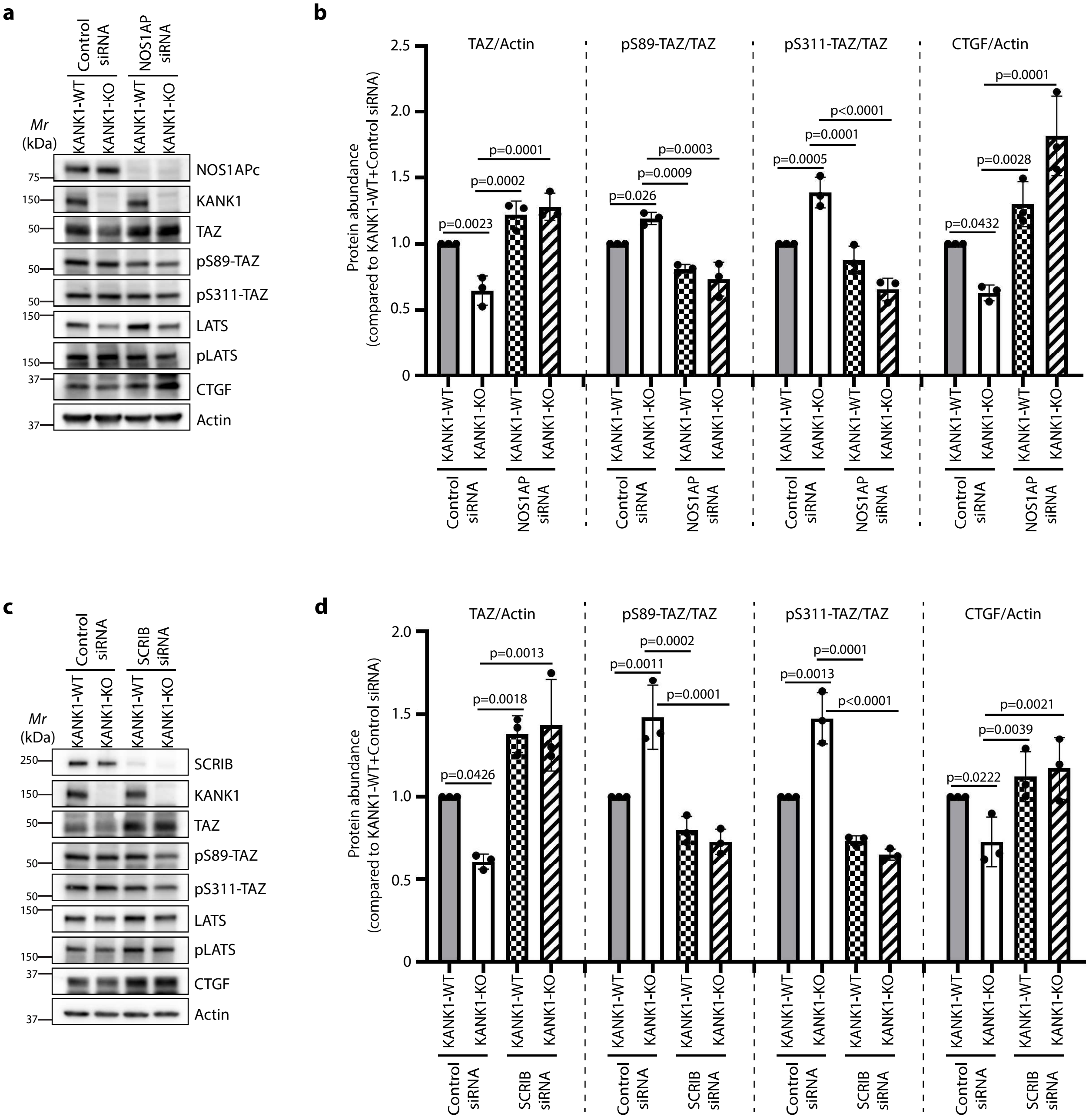

Supplementary Figure 12: Depletion of NOS1AP or SCRIB restores TAZ protein level in $MCF7^{KANK1\text{-}KO}$ cells

(a,b) Control- or NOS1AP-siRNA treated $MCF7^{KANK1\text{-}WT}$ and $MCF7^{KANK1\text{-}KO}$ cells were analyzed by WB with indicated antibodies. Representative blot from 3 independent experiments is shown in (**a**). The protein levels of TAZ, pS89-TAZ, pS311-TAZ and CTGF were quantified in (**b**). *P* values were calculated using two-way ANOVA with Dunnett's multiple comparisons test and are indicated on top of each comparison. Mean ± SD is shown. **(c,d)** Control siRNA or SCRIB siRNA treated $MCF7^{KANK1\text{-}WT}$ and $MCF7^{KANK1\text{-}KO}$ cells were analyzed by WB with indicated antibodies. Representative blot from 3 independent experiments is shown in (**c**). The protein levels of TAZ, pS89-TAZ, pS311-TAZ and CTGF were quantified in (d). *P* values were calculated using two-way ANOVA with Dunnett's multiple comparisons test and are indicated on top of each comparison. Mean ± SD is shown.

Source Data are provided as a Source Data file.

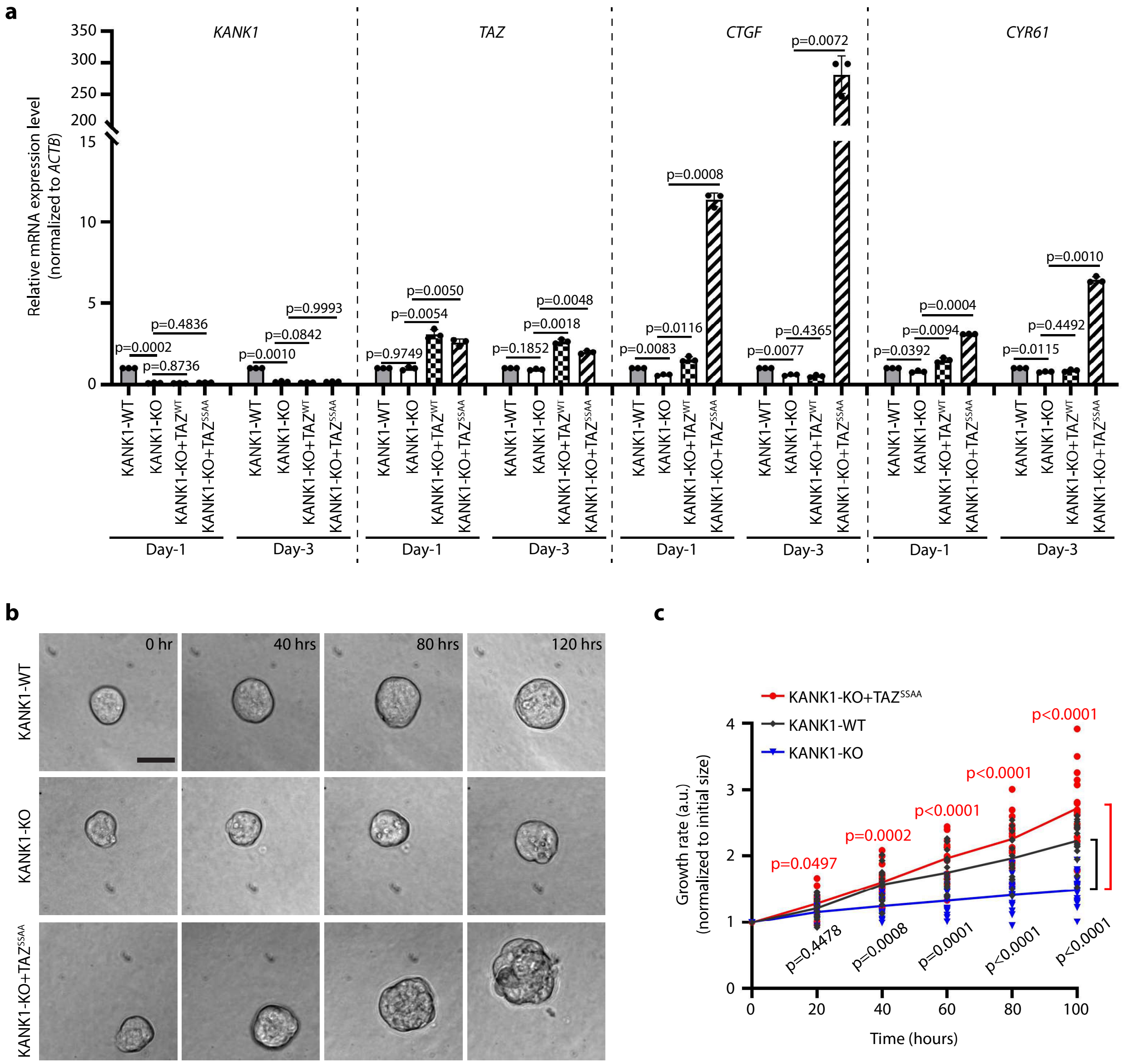

Supplementary Figure 13: TAZ stabilization rescues defects in MCF7$^{KANK1\text{-}KO}$ cells

(a) mRNA expression of *KANK1, TAZ, CTGF* and *CYR61* analyzed by qRT-PCR in MCF7$^{KANK1\text{-}WT}$, MCF7$^{KANK1\text{-}KO}$, MCF7$^{KANK1\text{-}KO+TAZ(WT)}$ and MCF7$^{KANK1\text{-}KO+TAZ(SSAA)}$ cells at day-1 and day-3 after overnight serum starvation. *ACTB* served as the internal control (n=3 biological replicates). *P* values were calculated using RM one-way ANOVA with Dunnett's multiple comparisons test and are indicated on top of each comparison. Mean ± SD is shown.
(b,c) MCF7$^{KANK1\text{-}WT}$, MCF7$^{KANK1\text{-}KO}$ and MCF7$^{KANK1\text{-}KO+TAZ(SSAA)}$ cells were embedded into Matrigel for a 5-day culture and monitored by time-lapse live-imaging. Still images from indicated time points are shown. Corresponding videos (#3-#5) are provided in Data Availability section. Quantification of growth rates of the tumoroids is shown in (**c**, n=16 for each condition). *P* values were calculated using two-way ANOVA with Dunnett's multiple comparisons test and are indicated on top of each comparison. Mean ± SD is shown.
Source Data are provided as a Source Data file.

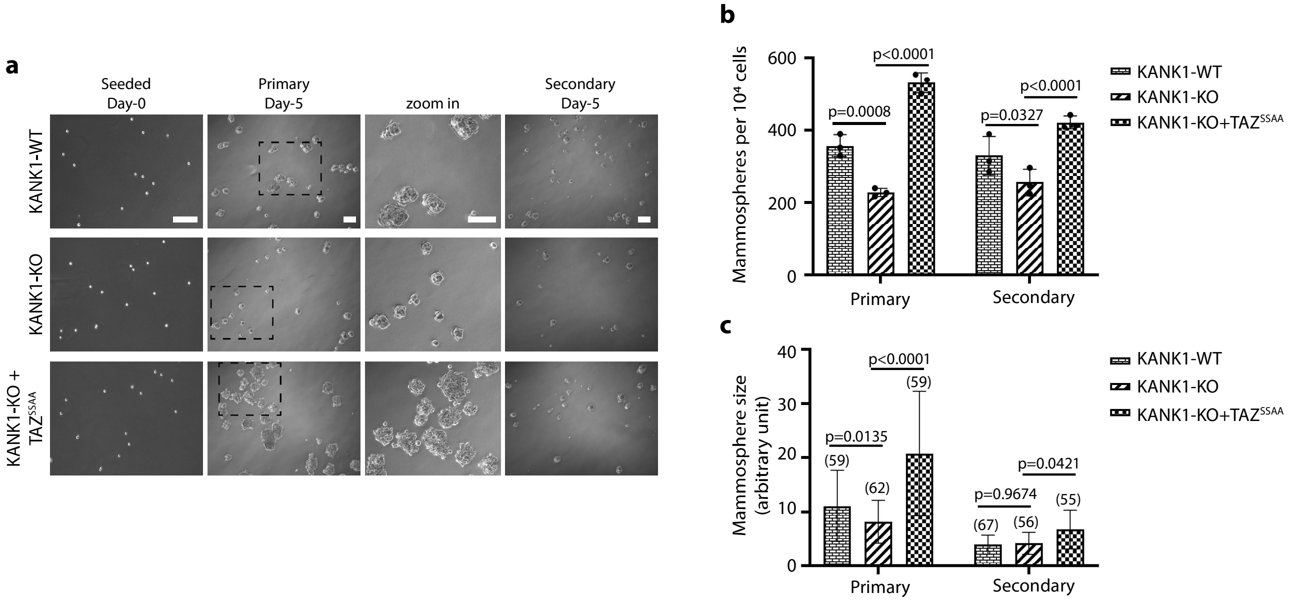

Supplementary Figure 14: TAZ stabilization rescues formation of mammospheres generated with MCF7$^{KANK1\text{-}KO}$ cells

(a-c) Representative images from 3 independent experiments of mammospheres formed at day 5 after aggregate induction for primary and secondary spheres (**a**). Scale bars: 200 µm. Mammosphere number (**b**) and size (**c**) were quantified. Numbers in brackets (**c**) indicate the numbers of spheres used to determine sphere size. *P* values were calculated using two-way ANOVA with Dunnett's multiple comparisons test and are indicated on top of each comparison. Mean ± SD is shown. Source Data are provided as a Source Data file.

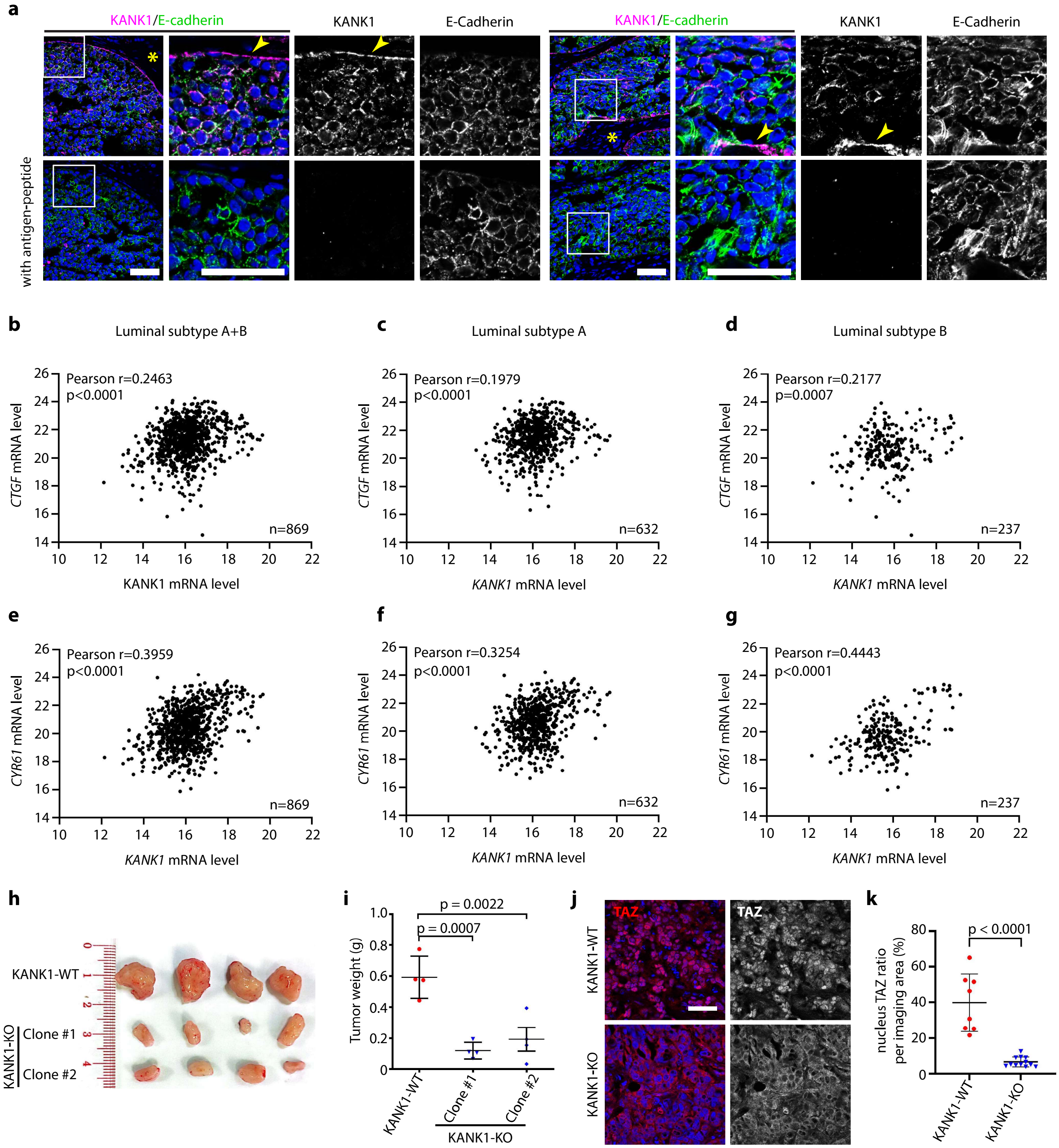

Supplementary Figure 15: Human breast cancer cell growth upon KANK1 loss

(a) Breast cancer tissues classified as luminal subtype A stained for KANK1 (magenta) and E-cadherin (green). KANK1 localizes at basal side (arrow heads) and cell-cell junctions of tumor cells. The tumor stroma (asterisks) is negative for KANK1 staining. Adjacent sections incubated with the antigen-peptide used for antibody generation served as KANK1 staining control. Scale bars: 50 µm. **(b-g)** Correlation of *KANK1* and *CTGF* (**b-d**), *KANK1* and *CYR61* (**e-g**) mRNA levels in the GDC TCGA BRCA patient cohort. Luminal subtypes were analyzed and presented with FPKM-UQ value. The number of patients included in the analysis is indicated on each graph. Pearson correlation coefficient (r) analysis and *P* value (*t*-distribution with n-2 degrees of freedom) calculation were performed in GraphPad Prism. **(h,i)** $MCF7^{KANK1\text{-}WT}$ and $MCF7^{KANK1\text{-}KO}$ cells were xenografted into the fat pad of immunodeficient mice. Tumors were dissected 8 weeks after injection, photographed (**h**) and weighed (**i**). *P* values were calculated using one-way ANOVA with Dunnett's multiple comparisons test and are indicated on top of each comparison. Mean ± SD is shown. **(j,k)** Tumors from (**h**) were sectioned and stained for TAZ (**j**). Nuclei were counterstained with DAPI. 2 samples from the KANK1-WT and 3 samples from KANK1-KO group were analyzed and quantified (n=4 independent imaging areas) (k). *P* value was calculated using unpaired Student's t test. Mean ± SD is shown. Scale bar: 50 µm.
Source Data are provided as a Source Data file.
