# Supplementary material for: KANK1 promotes breast cancer development by compromising Scribble-mediated Hippo activation
Source: Nat Commun. 2024 Nov 29;15:10381. doi: 10.1038/s41467-024-54645-9 (PMC11607453; doi:10.1038/s41467-024-54645-9)
Supplement: Supplementary file 2 — Description of Additional Supplementary Files [file 41467_2024_54645_MOESM2_ESM.pdf]

Description of Additional Supplementary Files

File name: Supplementary Data 1

Description: Antibody information

File name: Supplementary Data 2

Description: Plasmid information

File name: Supplementary Data 3

Description: qRT-PCR primers

File name: Supplementary Data 4

Description: Peptide sequence for antibody generation

File name: Supplementary Data 5

Description: PCR primers for amplification of the Cas9 targeting regions

File name: Supplementary Data 6

Description: Human sample information for correlation analysis

File name: Supplementary Movie 1

Description: KANK1-WT^{PyMT} tumoroid growth

File name: Supplementary Movie 2

Description: KANK1-KO^{PyMT} tumoroid growth

File name: Supplementary Movie 3

Description: MCF7$^{KANK1\text{-}WT}$ tumoroid growth

File name: Supplementary Movie 4

Description: MCF7$^{KANK1\text{-}KO}$ tumoroid growth

File name: Supplementary Movie 5

Description: MCF7$^{KANK1\text{-}KO}$ + TAZ^{SSAA} tumoroid growth
