# Supplementary material for: KANK1 promotes breast cancer development by compromising Scribble-mediated Hippo activation
Source: Nat Commun. 2024 Nov 29;15:10381. doi: 10.1038/s41467-024-54645-9 (PMC11607453; doi:10.1038/s41467-024-54645-9)
Supplement: Supplementary file 14 — Reporting Summary [file 41467_2024_54645_MOESM14_ESM.pdf]

nature portfolio

Corresponding author(s): Shiny Shengzhen Guo
Reinhard Fässler

Last updated by author(s): Nov 18, 2024

Reporting Summary

Nature Portfolio wishes to improve the reproducibility of the work that we publish. This form provides structure for consistency and transparency in reporting. For further information on Nature Portfolio policies, see our Editorial Policies and the Editorial Policy Checklist.

Statistics

For all statistical analyses, confirm that the following items are present in the figure legend, table legend, main text, or Methods section.

n/a	Confirmed	
☐	☒	The exact sample size (*n*) for each experimental group/condition, given as a discrete number and unit of measurement
☐	☒	A statement on whether measurements were taken from distinct samples or whether the same sample was measured repeatedly
☐	☒	The statistical test(s) used AND whether they are one- or two-sided<br>*Only common tests should be described solely by name; describe more complex techniques in the Methods section.*
☐	☒	A description of all covariates tested
☐	☒	A description of any assumptions or corrections, such as tests of normality and adjustment for multiple comparisons
☐	☒	A full description of the statistical parameters including central tendency (e.g. means) or other basic estimates (e.g. regression coefficient) AND variation (e.g. standard deviation) or associated estimates of uncertainty (e.g. confidence intervals)
☐	☒	For null hypothesis testing, the test statistic (e.g. *F*, *t*, *r*) with confidence intervals, effect sizes, degrees of freedom and *P* value noted<br>*Give P values as exact values whenever suitable.*
☒	☐	For Bayesian analysis, information on the choice of priors and Markov chain Monte Carlo settings
☒	☐	For hierarchical and complex designs, identification of the appropriate level for tests and full reporting of outcomes
☐	☒	Estimates of effect sizes (e.g. Cohen's *d*, Pearson's *r*), indicating how they were calculated

Our web collection on statistics for biologists contains articles on many of the points above.

Software and code

Policy information about availability of computer code

Data collection	Zeiss (Jena) LSM780 confocal laser scanning microscope for immunofluorecence stainings<br>QExactive HF mass spectrometer (Thermo Fisher Scientific) or timsTOF Pro mass spectrometer (Bruker Daltonics) for proteomics<br>Nikon Eclipase Ti2 for live-imaging<br>IVIS-100 Xenogen, PerkinElmer for luciferase-tumor imaging<br>LightCycler480 Instrument II (Roche) for qRT-PCR<br>FACSAria Cell Sorter (BD) for GFP/mCherry cells enrichement<br>Amersham Imager 600 for western blots
Data analysis	GraphPad Prism (version 9.0) was used for statistical analysis.<br>Fiji (Image J) was used for quantification of immunoblot bands, immunofluorescence and live-cell imaging signals.<br>Maxquant (version 2.0.1.0) was used for processing raw proteomic data.<br>Perseus software (version 1.5.5.3) was used to analyze proteomic data.

For manuscripts utilizing custom algorithms or software that are central to the research but not yet described in published literature, software must be made available to editors and reviewers. We strongly encourage code deposition in a community repository (e.g. GitHub). See the Nature Portfolio guidelines for submitting code & software for further information.

Data

Policy information about availability of data

All manuscripts must include a data availability statement. This statement should provide the following information, where applicable:

- Accession codes, unique identifiers, or web links for publicly available datasets
- A description of any restrictions on data availability
- For clinical datasets or third party data, please ensure that the statement adheres to our policy

The mass spectrometry proteomics data have been deposited to the ProteomeXchange Consortium via the PRIDE partner repository with the dataset identifier PXD049122 (http://www.ebi.ac.uk/pride/archive/projects/PXD049122). All other supporting data are deposited into the Data Availability section, which include the uncropped blots of Fig. 4c, 4e, 5b, 5e, 7a, 7c, 8a, 8c, 8e, Supplementary Fig. 1b, 6e, 7a, 7b, 9a, 10b, 11e, 12a and 12c; the source data for statistical analysis or plotting of Fig. 1a-f, 2a-c, 2e, 3a,b,e,f, 4d, 4f, 5c, 5f, 6b, 7b, 7d, 8b, 8d, 8f-i, 9b, 9e, 10b and 10d, Supplementary Fig. 1c, 3b, 4a, 4d, 6d, 9c, 10a, 10c, 11b, 11d, 11f, 12b, 12d, 13a, 13c, 14b, 14c, 15b-g, 15i and 15k in excel file format; the FACS data and the videos for Fig. 3d and Supplementary Fig. 11b.

Research involving human participants, their data, or biological material

Policy information about studies with human participants or human data. See also policy information about sex, gender (identity/presentation), and sexual orientation and race, ethnicity and racism.

Reporting on sex and gender	All breast cancer tissues analyzed were from female patients of Caucasican background.
Reporting on race, ethnicity, or other socially relevant groupings	All breast cancer tissues analyzed were from female patients of Caucasican background. Written consent for usage of patient tumor tissues for research purpose was provided by the patients or their family members, which was approved by the Ethics Committee of the University of Cologne (#13-091).
Population characteristics	All breast cancer tissues analyzed were from females of Caucasian background.
Recruitment	Written consent for usage of patient tumor tissues for research purpose was provided by the patients or their family members, which was approved by the Ethics Committee of the University of Cologne (#13-091).
Ethics oversight	Written consent for usage of patient tumor tissues for research purpose was provided by the patients or their family members, which was approved by the Ethics Committee of the University of Cologne (#13-091).

Note that full information on the approval of the study protocol must also be provided in the manuscript.

Field-specific reporting

Please select the one below that is the best fit for your research. If you are not sure, read the appropriate sections before making your selection.

☒ Life sciences ☐ Behavioural & social sciences ☐ Ecological, evolutionary & environmental sciences

For a reference copy of the document with all sections, see nature.com/documents/nr-reporting-summary-flat.pdf

Life sciences study design

All studies must disclose on these points even when the disclosure is negative.

Sample size	The sample sizes were determined based on experience on the reliable measurement of the experimental results according to the standards in previous publications (Roswall et al., Nature Medicine, 2018; Chen et al., Genes&Development, 2014; Zhan et al., Cell, 2008; Chen et al., NCB, 2022) in the field and sufficient to represent the significance of difference in different conditions. No statistical method was used to predetermine the sample size.
Data exclusions	No data was excluded in this study.
Replication	The sample size of each experiment and the number of experimental replicates were indicated in figure legends or methods. All experiments were successfully replicated.
Randomization	Samples were allocated into KANK1-WT or KANK1-KO groups in terms of the genotyping/westerb blot results for both animals and cancer cells. Data for comparisons of KANK1 localization at cell-cell contacts were acquired by randomly taking images of clustered cells in the well or on the coverslips. Statistical data for quantification was collected over months or years, thus ensure reproducibility despite being gathered from different passages.
Blinding	For KANK1-TAZ correlation in tumor tissues from both mouse and human, carcinoma in situ regions were imaged to show KANK1 expression at both basement membrane and at cell-cell contacts. Image acquisition from different cell lines or at different conditions were not blinded but relied on unbiased data collection from random regions in the wells or coverslips.

Reporting for specific materials, systems and methods

We require information from authors about some types of materials, experimental systems and methods used in many studies. Here, indicate whether each material, system or method listed is relevant to your study. If you are not sure if a list item applies to your research, read the appropriate section before selecting a response.

Materials & experimental systems

n/a	Involved in the study	
☐	☒	Antibodies
☐	☒	Eukaryotic cell lines
☒	☐	Palaeontology and archaeology
☐	☒	Animals and other organisms
☒	☐	Clinical data
☒	☐	Dual use research of concern
☒	☐	Plants

Methods

n/a	Involved in the study	
☒	☐	ChIP-seq
☒	☐	Flow cytometry
☒	☐	MRI-based neuroimaging

Antibodies

Antibodies used

The following antibodies were used for western blotting (WB) and/or immunofluorescence (IFC):
home-made KANK1, rabbit, 1:1000 for WB and 1:2000 for IFC; KANK1 (Sigma, HPA056090), rabbit, 1:1000 for both WB and IFC; home-made KANK2, rabbit, 1:1000 for WB and 1:4000 for IFC; home-made KANK3, rabbit, 1:6000 for IFC; home-made KANK4, rabbit, 1:1000 for IFC; active caspase-3 (cell signaling, #9661), rabbit, 1:200 for IFC; Cyclin D1 (cell signling, 2978S), rabbit, 1:600 for IFC; Phospho-histone 3(Upstate, 16-189), rabbit, 1:600 for IFC; Nidogen (Millipore, MAB1946-I), rat, 1:2000 for IFC; FITC-SMA (Sigma, F3777), mouse, 1:2000 for IFC); SMA (Sigma, A2547), mouse 1:2000 for IFC; Paxillin (Transduction Laboratories, 610051), mouse, 1:800 for IFC; E-cadherin (ThermoFisher, 13-1900), rat, 1:800 for IFC; Pan-cadherin (Santa Cruz, sc-1499), goat, 1:600 for IFC; Ki67 (Abcam, ab15580), rabbit, 1:600 for IFC; Alexa-Fluor 647 integrin beta1 (BioLegend, #303018), mouse, 1:400 for IFC; Talin-1 (Bio-Rad, MCA4770), mouse, 1:200 for IFC; Talin-2 (Abcam, ab105458), mouse 1:200 for IFC; KIF21A (Proteintech, 27276-1-AP), rabbit, 1:1000 for WB; KIF21A (gift from Professor Engle Elizabeth), rabbit, 1:1000 for WB; NOS1AP (Sigma, HPA030066), rabbit, 1:1000 for WB; home-made pan-NOS1AP, rabbit, 1:1000 for WB and 1:2000 for IFC; a isoform specific NOS1AP (home-made), rabbit, 1:1000 for both WB and IFC; c isoform specific NOS1AP (home-made), 1:1000 for WB; c isoform specific antibody (generated in James Fawcett's lab), rabbit, 1:2000 for IFC; Scribble (GeneTex, GTX107692), rabbit, 1:1000 for WB; YAP/TAZ (cell signaling, #8418), rabbit, 1:1000 for WB and 1:800 for IFC; TAZ (Sigma, HPA007415), rabbit, 1:1000 for both WB and IFC; S89-TAZ (cell signaling, #59971), rabbit, 1:1000 for WB; S311-TAZ (ThermoFisher, #712011), rabbit, 1:1000 for WB; CTGF (cell signaling, #86641), rabbit, 1:1000 for WB; LATS2 (Abcam, ab243657), rabbit, 1:1000 for WB; T1041-LATS2 (gift from Dr Norikazu Yabuta), rabbit, 1:1000 for WB; Actin (Sigma, A5441), mouse, 1:2000 for WB; GAPDH (Millipore, CB1001), mouse, 1:2000 for WB; Tubulin (Millipore, MAB1864), rat, 1:2000 for WB; GFP (ThermoFisher, A10262), chicken, 1:1000 for WB; mCherry (SICGEN, AB0040-200), goat, 1:1000 for WB; anti-rabbit Cy3 (Jackson Lab, 711-165-152), donkey, 1:800 for IFC; anti-rat Alexa 488 (ThermoFisher, A21208), donkey, 1:800 for IFC; anti-mouse Alexa 488 (ThermoFisher, A21202), donkey, 1:800 for IFC; anti-goat Alexa 647(ThermoFisher, A21447), 1:500 for IFC; anti-mouse Alexa 647 (ThermoFisher, A31571), 1:500 for IFC; anti-rat Alexa 647 (Jackson Lab, 712-605-153), 1:500 for IFC; anti-rabbit HRP (Jackson Lab, 711-035-152), 1:1000 for WB; anti-mouse HRP (Jackson Lab, 715-035-151), 1:1000 for WB; anti-rat HRP (Jackson Lab, 712-035-150), 1:1000 for WB; anti-goat HRP (Jackson Lab, 705-035-147), 1:1000 for WB; anti-chicken HRP (Jackson Lab, 703-035-155), 1:1000 for WB.

Validation

The references of the usage of the antibodies listed above are as follows:
home-made NOS1AP and NOS1AP (Sigma, HPA030066) antibodies were verified in the current work with NOS1AP knockout cells; home-made KANK antibodies, KANK1 (Sigma, HPA056090), Pan-cadherin (Santa Cruz, sc-1499), Nidogen (Millipore, MAB1946-I), Tubulin (Millipore, MAB1864), SMA (Sigmanti-mouse Alexa 488 (ThermoFisher, A21202), a, A2547), anti-rabbit Cy3 (Jackson Lab, 711-165-152), anti-rat Alexa 488 (ThermoFisher, A21208), anti-rat Alexa 647 (Jackson Lab, 712-605-153), anti-goat Alexa 647 and anti-mouse Alexa 647 (ThermoFisher, A31571) in Guo et al., Exp Cell Res, 2021; Paxillin (Transduction Laboratories, 610051), Cyclin D1 (cell signling, 2978S) and GAPDH (Millipore, CB1001) in Chen et al., NCB, 2022; active caspase-3 (cell signaling, #9661) in Guo et al., Development, 2020; Phospho-histone 3(Upstate, 16-189) in Stevens, Sci Rep, 2017; E-cadherin (ThermoFisher, 13-1900) in Sun et al, EMBO J., 2022; Ki67 (Abcam, ab15580) in Laraba et al., Brain, 2023; Alexa-Fluor 647 integrin beta1 (BioLegend, #303018) in Häsler et al., J Cell Biol, 2020; Talin-1 (Bio-Rad, clone97H6, MCA4770) and Talin-2 (Abcam, clone 68E7, ab105458) in Praekelt et al., Eur J Cell Biol, 2012; KIF21A (gift from Professor Engle Elizabeth) in Tischfield et al., Cell, 2010; Scribble (GeneTex, GTX107692) in Youk et al., Cell Stem Cell, 2020; S89-TAZ (cell signaling, #59971), TAZ (Sigma, HPA007415) and YAP/TAZ (cell signaling, #8418) in Want et al., Nat Commun, 2020; S311-TAZ (ThermoFisher, #712011) and KIF21A (Proteintech, 27276-1-AP) were verified by the producer; CTGF (cell signaling, #86641) in Zhao et al., Int J Nanomedicine, 2018; LATS2 (Abcam, ab243657) in Wu et al., Mol Carcinog, 2023; T1041-LATS2 (gift from Dr Norikazu Yabuta) in Zhang et al., EMBO J, 2012; Actin (Sigma, A5441) in Li et al., Cell Biosci, 2019; GFP (ThermoFisher, A10262) in Grødem et al., Nat Commun, 2023; mCherry (SICGEN, AB0040-200) in Wu et al., Elife, 2020; Secondary antibodies for WB: anti-rabbit HRP (711-035-152), anti-rabbit HRP (711-035-152), anti-mouse HRP (715-035-151), anti-rat HRP (712-035-150), anti-goat HRP (705-035-147) and anti-chicken HRP (703-035-155) were all from Jackson Lab.

Eukaryotic cell lines

Policy information about cell lines and Sex and Gender in Research

Cell line source(s)

MCF7 cell ATCC number: HTB-22

	HEK293T cell ATCC number: CRL-3216
Authentication	MCF7 and HEK293T cell lines were purchased from ATCC and not authenticated in the study.
Mycoplasma contamination	All cell lines used were tested negative for mycoplasma contamination.
Commonly misidentified lines (See ICLAC register)	Not present in this study

Animals and other research organisms

Policy information about studies involving animals; ARRIVE guidelines recommended for reporting animal research, and Sex and Gender in Research

Laboratory animals	The MMTV-PyMT (stock NO. 022974) mouse strain was obtained from the Jackson Laboratory. KANK1-knockout (KANK1-KO) mouse strain was generated by crossing the KANK1-flox mouse with the transgenic line carrying a uniquitous expression of Cre recombinase (Tg (Nes-cre)1Wme), which was maintained at the Max-Planck Institute of Biochemistry, Martinsreid, Germany.
Wild animals	Wild-type (WT) C57BL/6 NRjMpi was originally obtained from Janvier Labs, and was maintained in the animal facility at the Max-Planck Institute of Biochemistry, Martinsried, Germany.
Reporting on sex	Mammary glands dissected out for KANK1 expression profiling at different life phases (virgin, pregnancy, lactation and involution) were from female mice.<br>Breast cancer development was analyzed on female mice. Male mice with MMTV-PyMT oncogene and KANK1 allele heterozygous was used for generating the litter mates.
Field-collected samples	All experimental animals were housed in rooms with strict barriers at a temperature of 22 ± 1.5oC, with the humidity at 55± 5% and with artificial lighting with the dark cycle of 14:10 hours. Animals were housed in autoclaved cages with filtered wood shaving as the bedding, with autoclaved water and commercial standard diet for mice. Animal staffs and scientists are protected with sterilized clothing and shoes, gloves, face masks and bonnets.<br>And the end-of-experiment was set as follows: moderate score for longer than 24h, e.g. single tumor (diameter<1,5 cm) or several tumors (diameter in sum < 3 cm) in combination with other symptoms. Kill immediately if single tumor of (diameter ≥1,5cm) or several tumors (diameter in sum ≥3cm). Endpoints according to scoresheet: total score >7 or single score 3.
Ethics oversight	All mouse experiments were performed according to the regulations approved by the government of Upper Bavaria, Germany (license NO: 55.2-2532.Vet 02-20-182), and the Committee on the Use of Live Animals in Teaching and Research of the University of Hong Kong (CULATR NO: 5842-21).

Note that full information on the approval of the study protocol must also be provided in the manuscript.

Plants

Seed stocks	not applicable for this study
Novel plant genotypes	not applicable for this study
Authentication	not applicable for this study
