# Supplementary material for: KANK1 promotes breast cancer development by compromising Scribble-mediated Hippo activation
Source: Nat Commun. 2024 Nov 29;15:10381. doi: 10.1038/s41467-024-54645-9 (PMC11607453; doi:10.1038/s41467-024-54645-9)
Supplement: Supplementary file 16 — Source Data [file 41467_2024_54645_MOESM16_ESM.zip › Source Data/Uncropped blots.pdf]

Fig. 4c

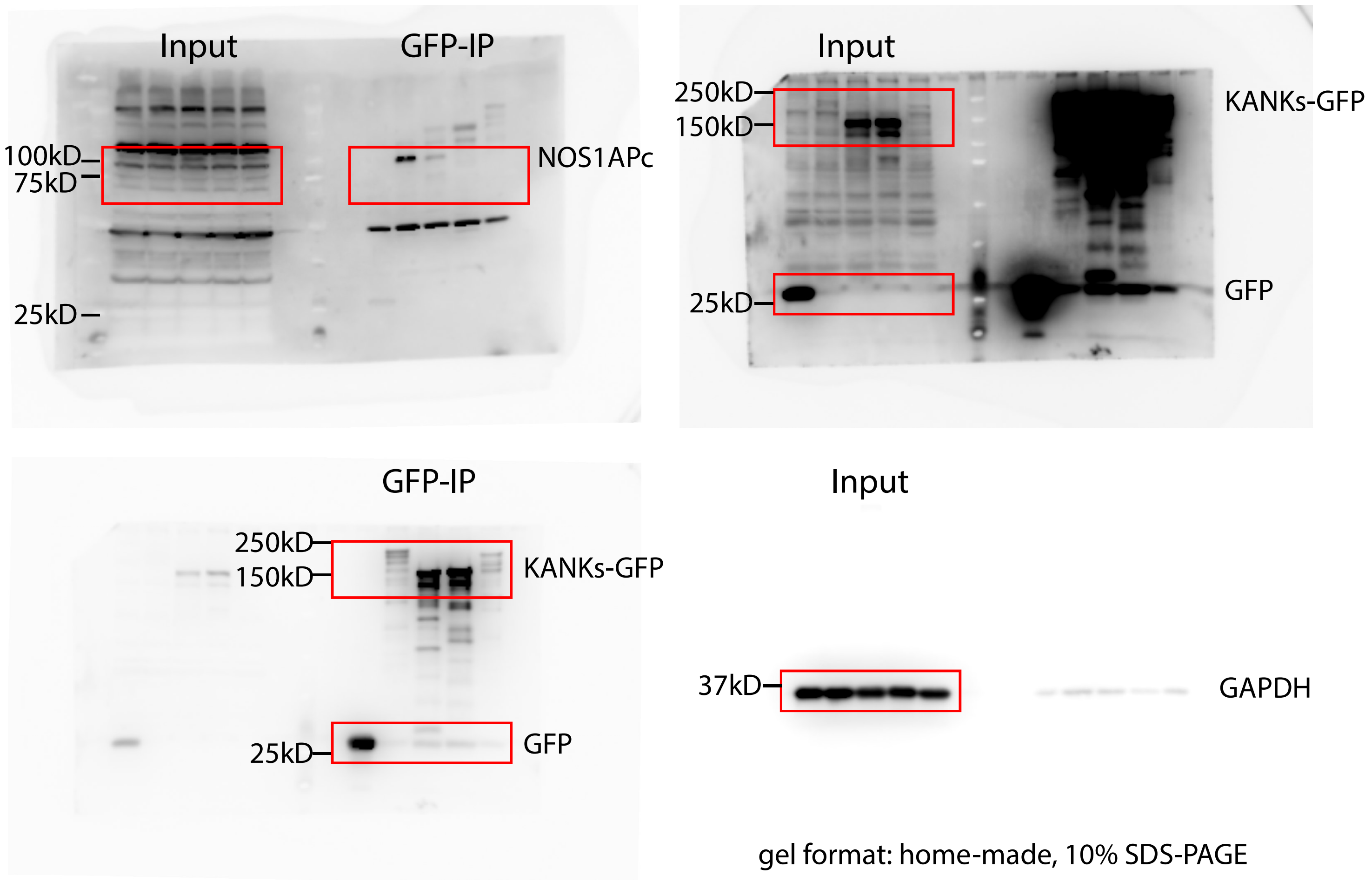

gel format: home-made, 10% SDS-PAGE

Fig. 4e

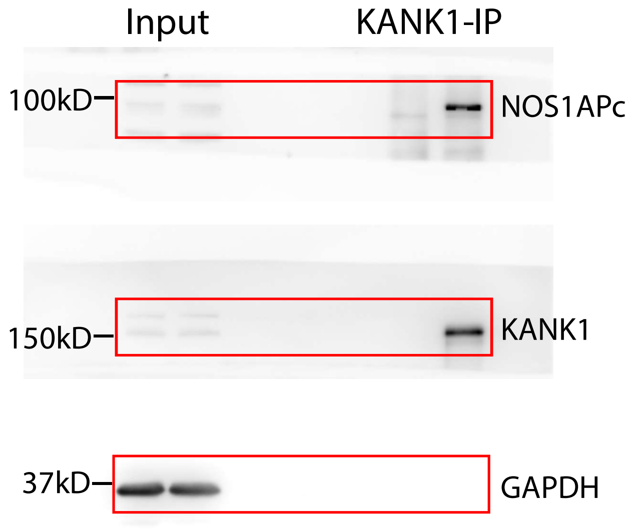

gel format: home-made, 10% SDS-PAGE

Fig. 5b

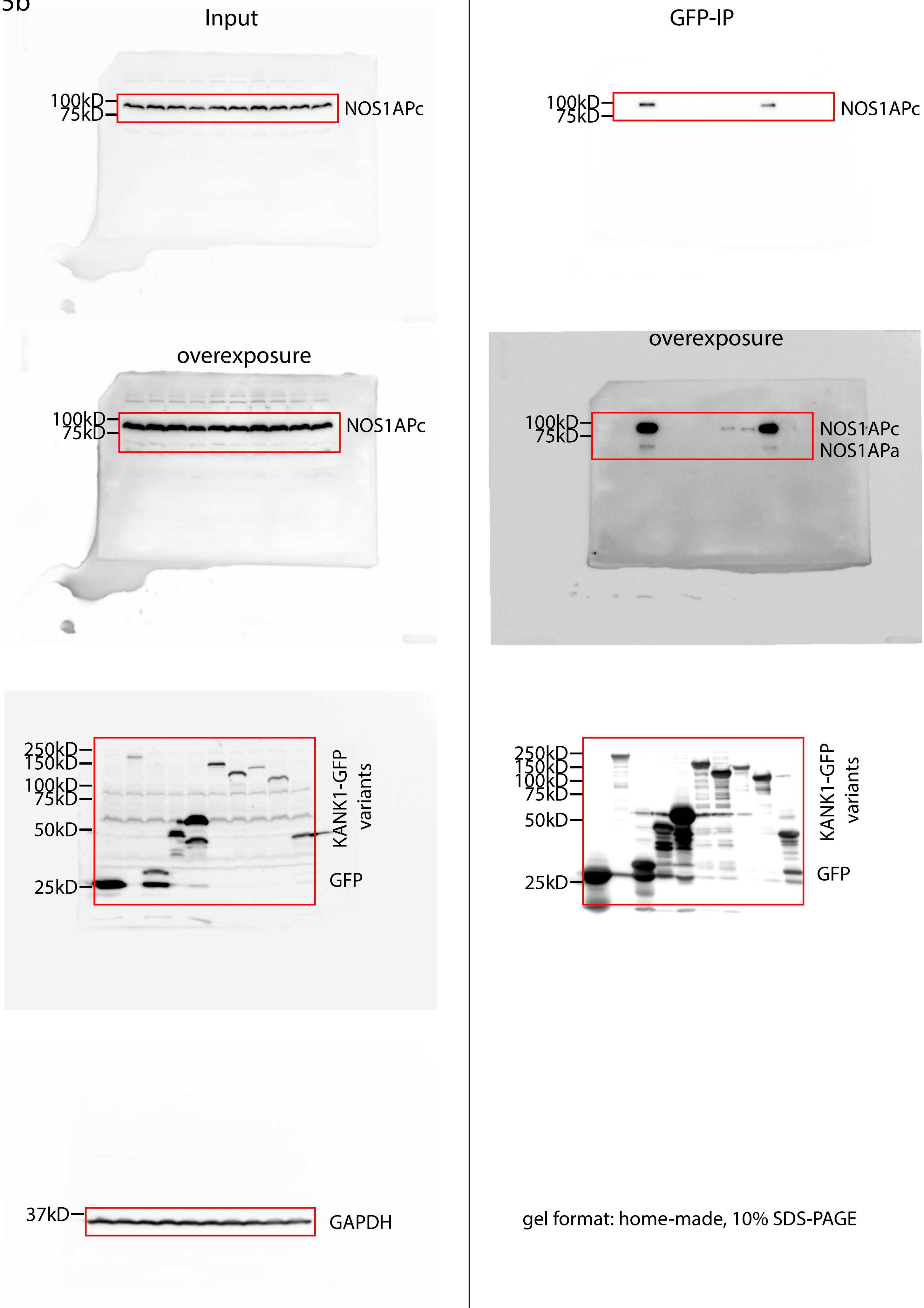

gel format: home-made, 10% SDS-PAGE

Fig. 5e

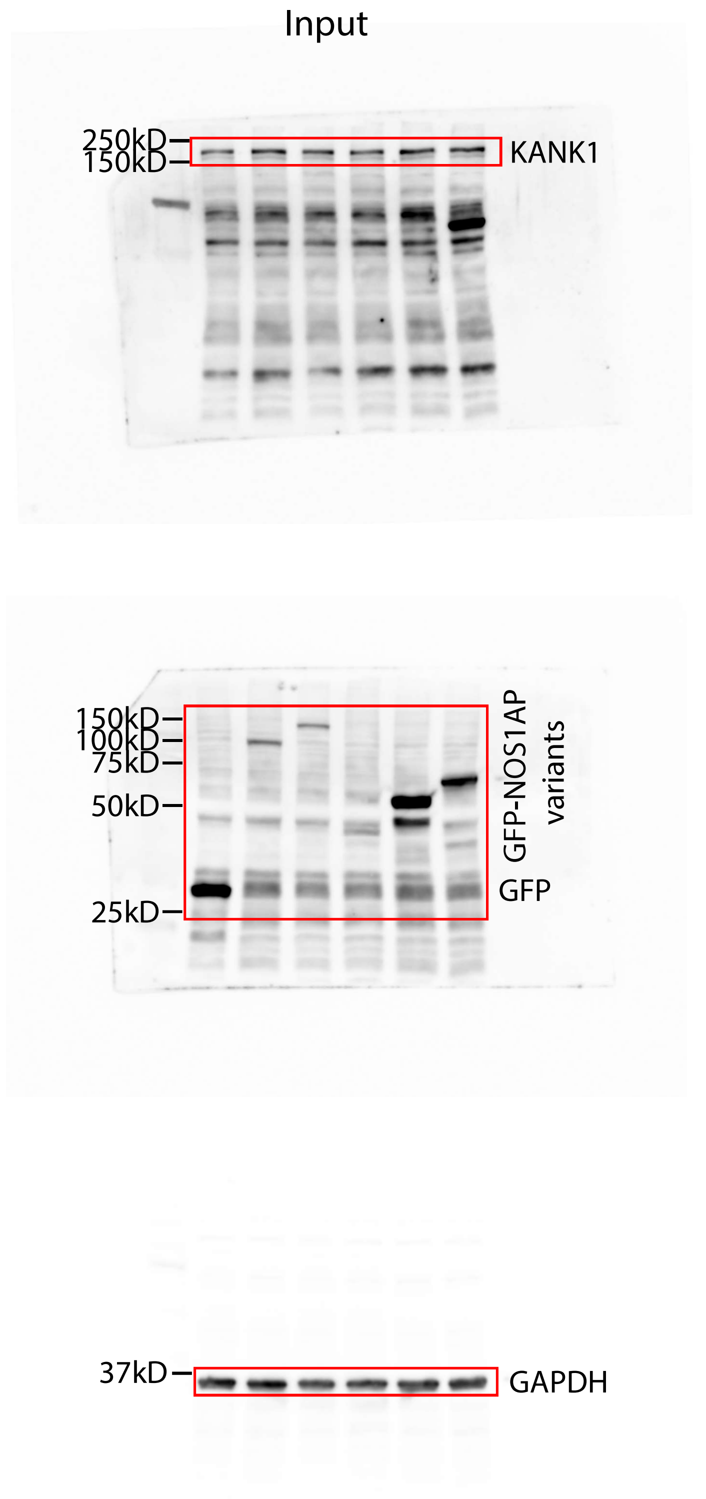

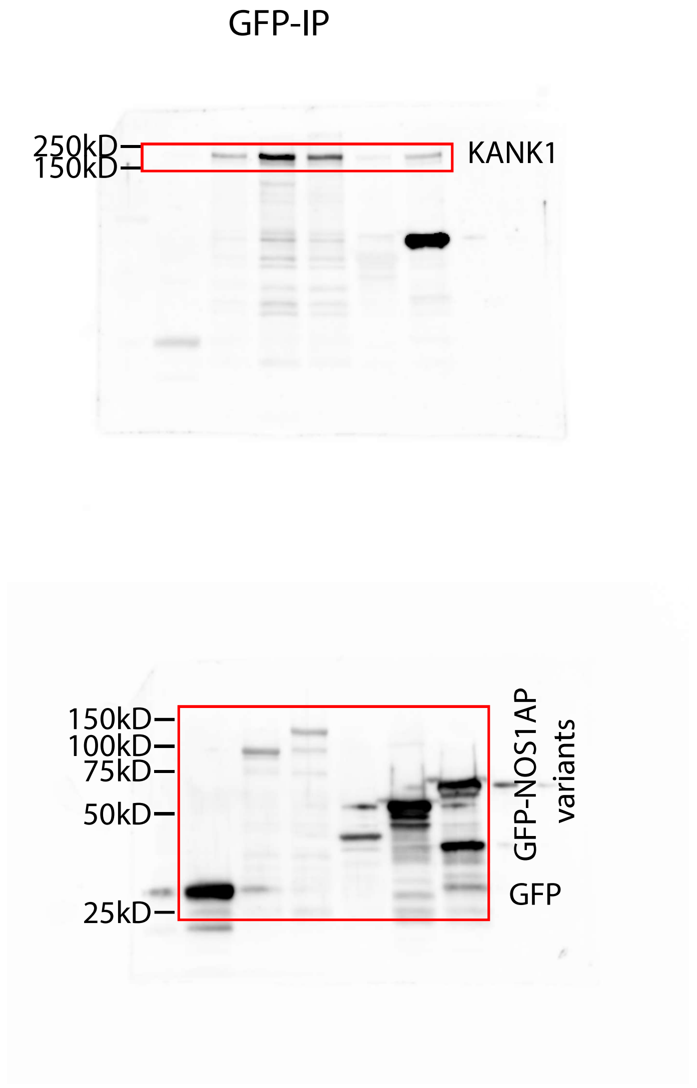

gel format: home-made, 10% SDS-PAGE

Fig. 7a

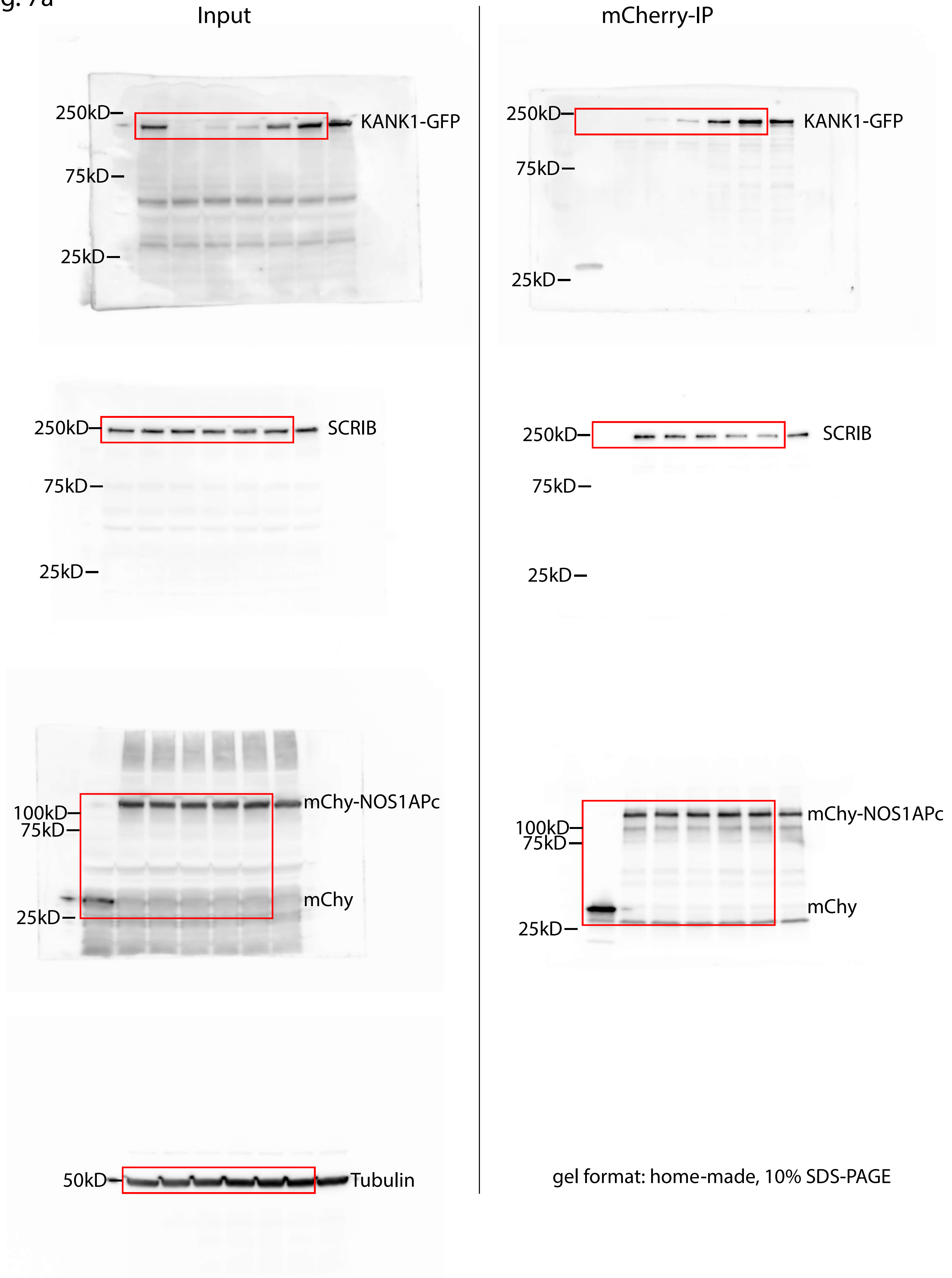

Input
mCherry-IP
250kD
75kD
25kD
KANK1-GFP
SCRIB
100kD
mChy-NOS1APc
mChy
50kD
Tubulin
gel format: home-made, 10% SDS-PAGE

Fig. 7c

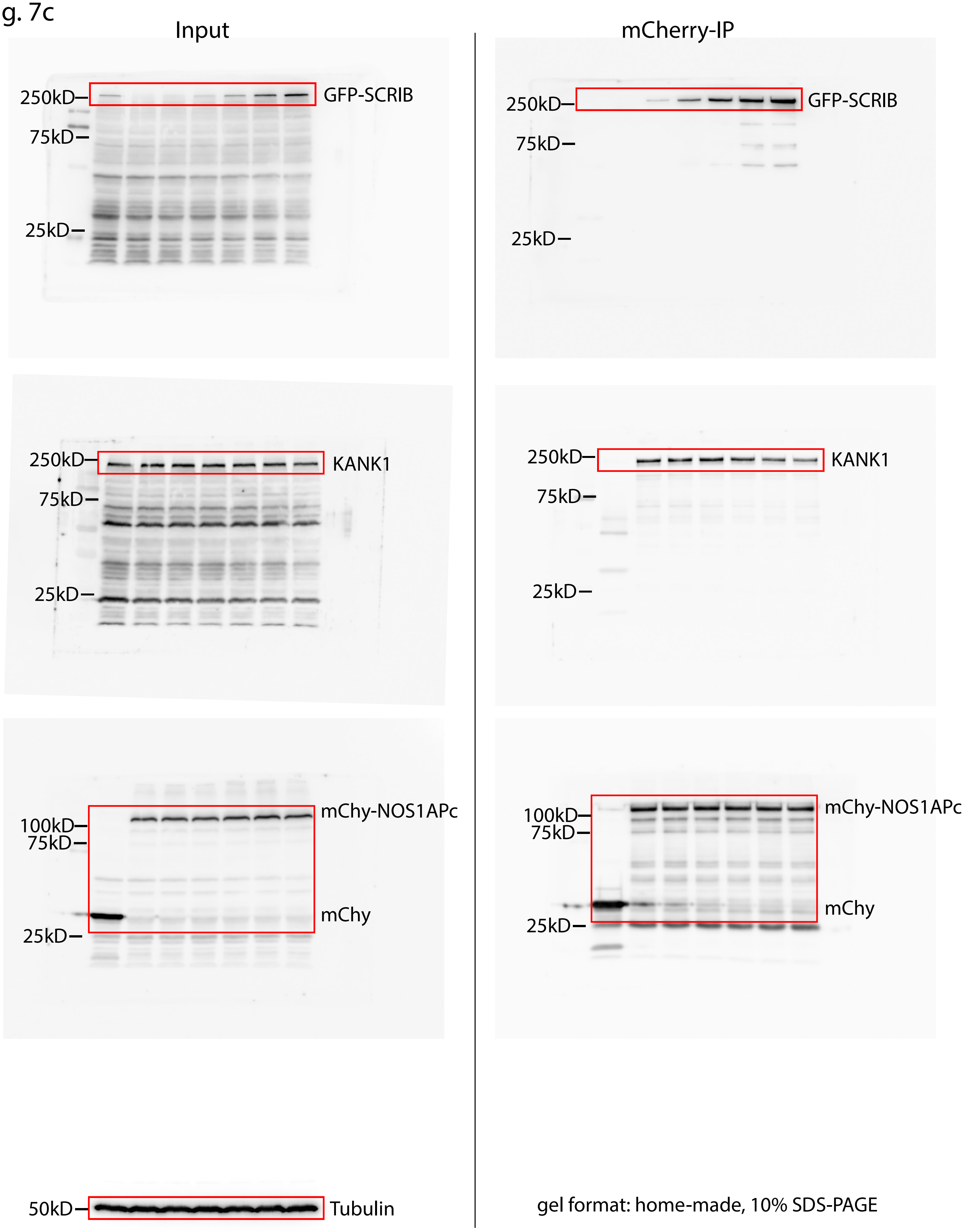
Input
mCherry-IP
250kD
75kD
25kD
GFP-SCRIB
250kD
75kD
25kD
GFP-SCRIB
250kD
75kD
25kD
KANK1
250kD
75kD
25kD
KANK1
100kD
75kD
25kD
mChy-NOS1APc
mChy
100kD
75kD
25kD
mChy-NOS1APc
mChy
50kD
Tubulin
gel format: home-made, 10% SDS-PAGE

Fig. 8a

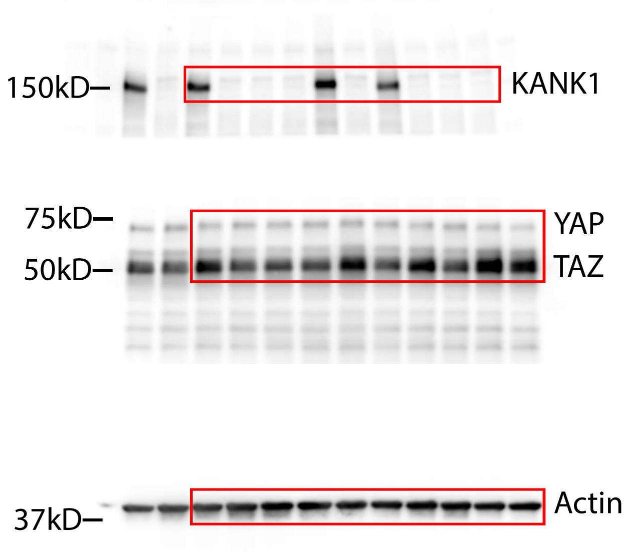

Fig. 8c

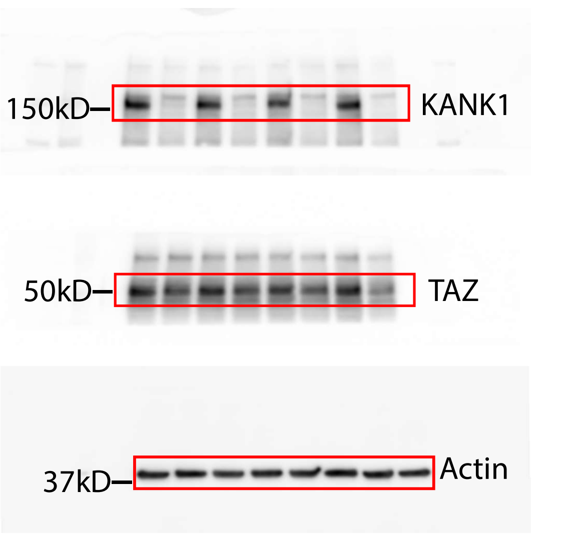

gel format: Bio-Rad (#4561086), gradient (4-15%) SDS-PAGE

Fig. 8e

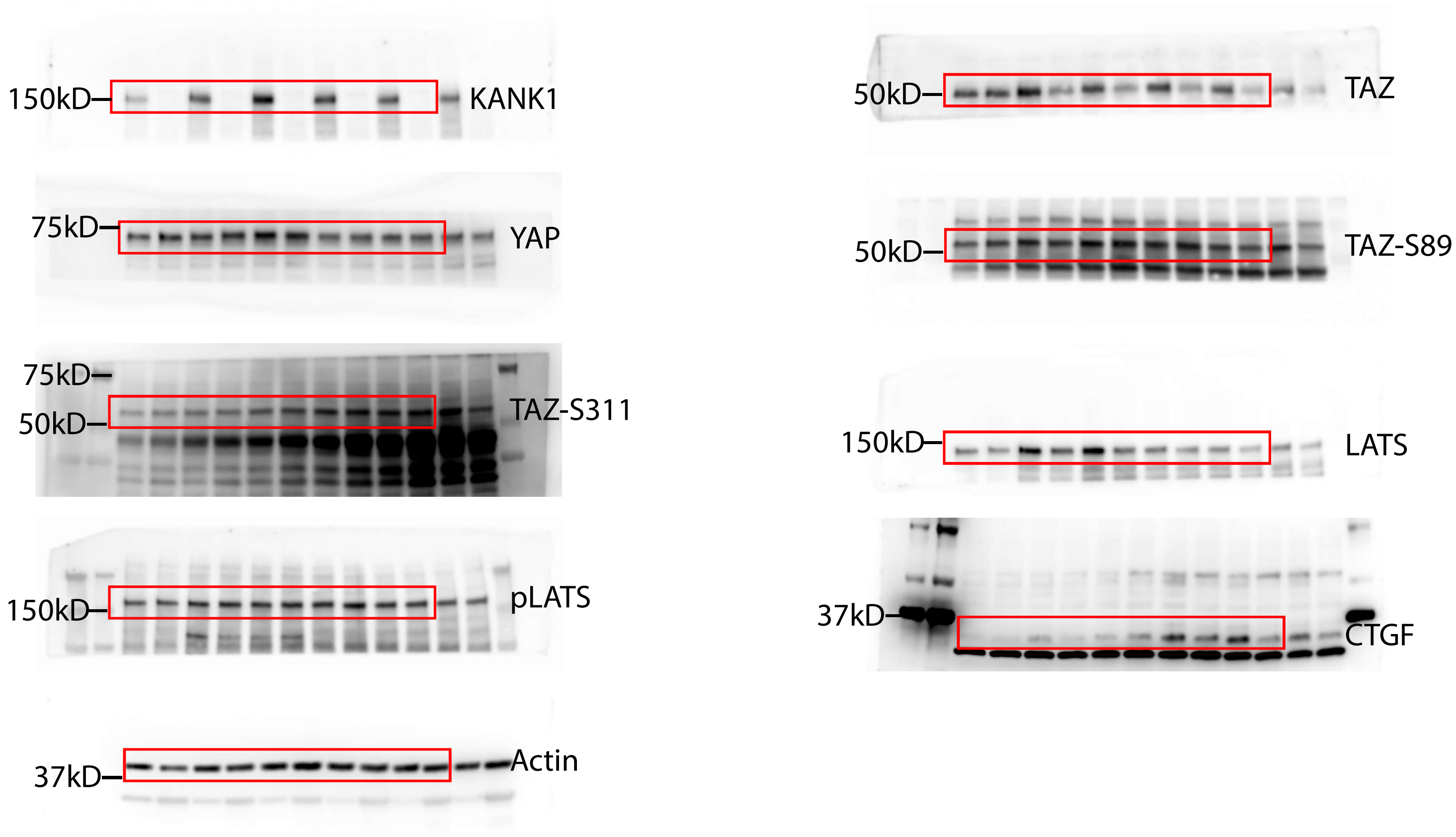

gel format: Bio-Rad (#4561086), gradient (4-15%) SDS-PAGE

Supplementary Fig. 1b

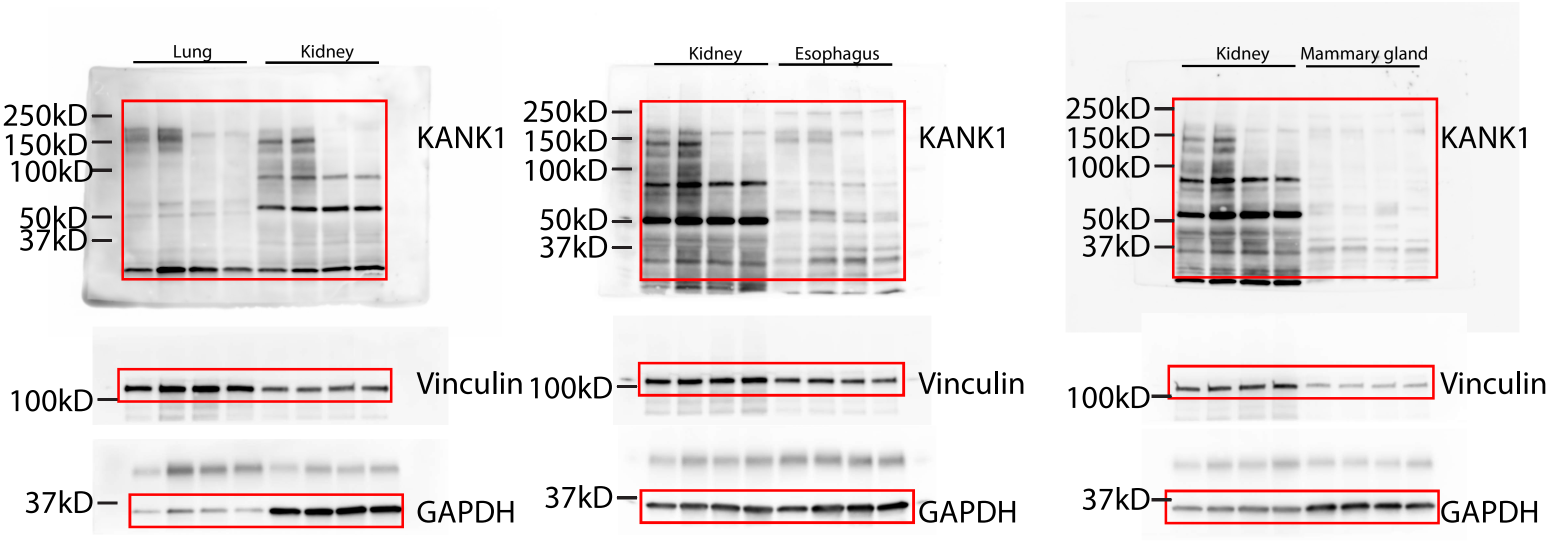

gel format: Bio-Rad (#4561083), gradient (4-15%) SDS-PAGE

Supplementary Fig. 6e

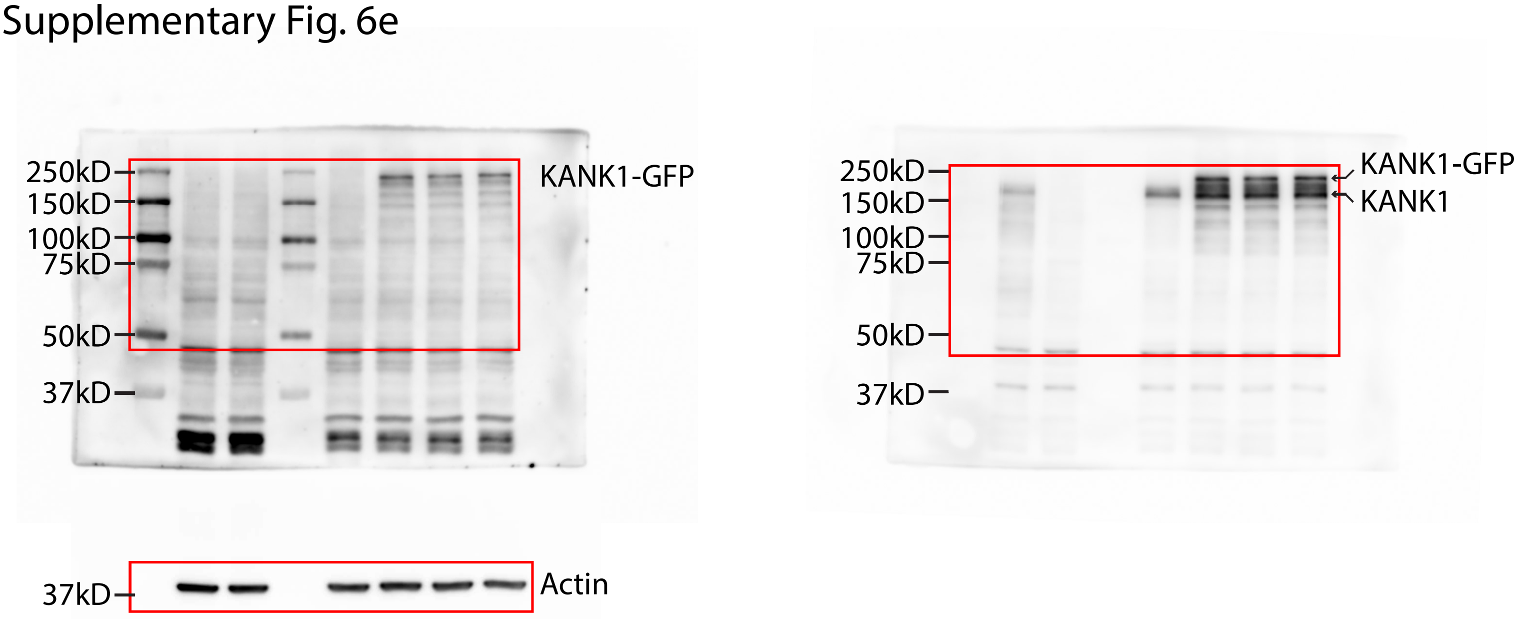

gel format: Bio-Rad (#4561083), gradient (4-15%) SDS-PAGE

Supplementary Fig. 7a

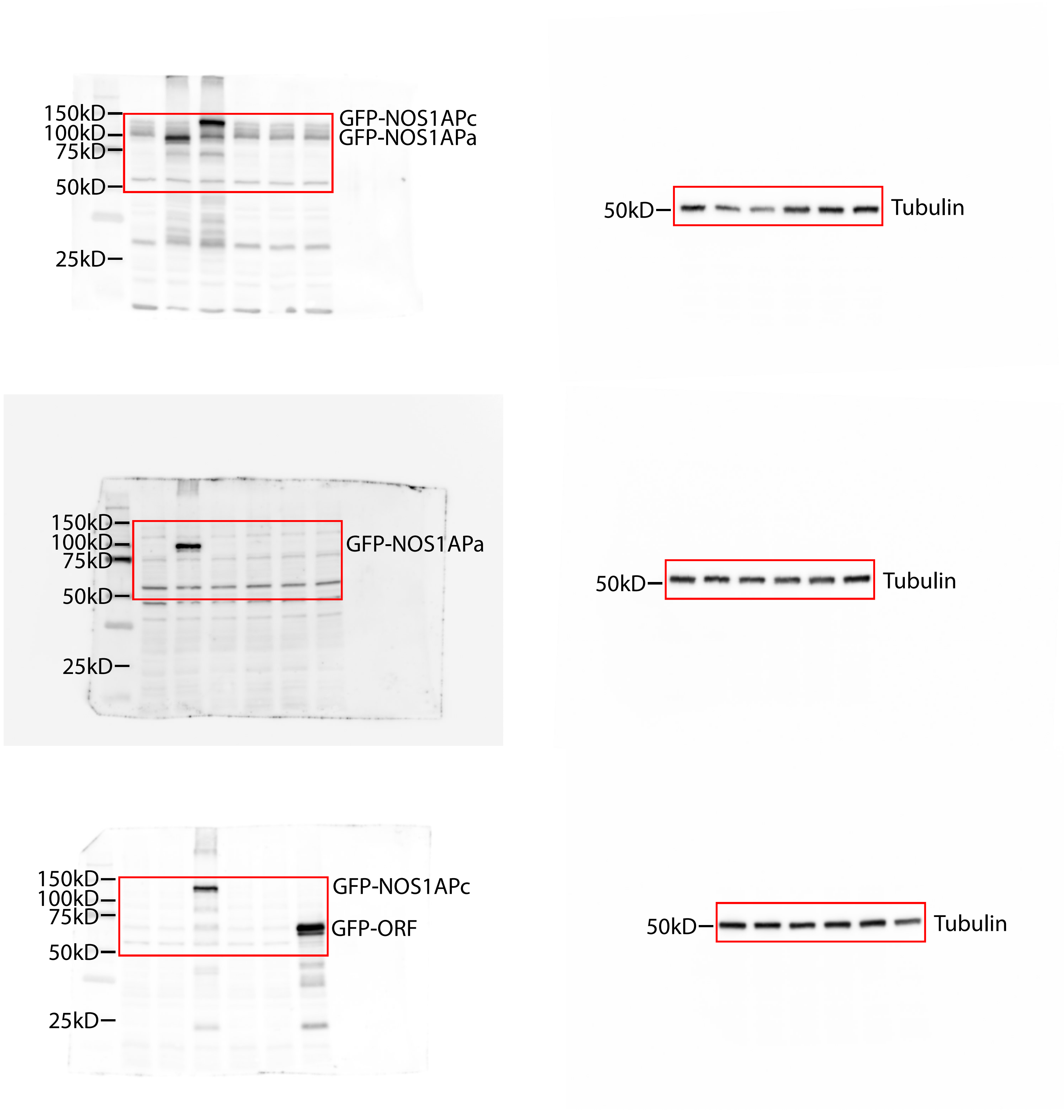

gel format: home-made, 10% SDS-PAGE

Supplementary Fig. 7b

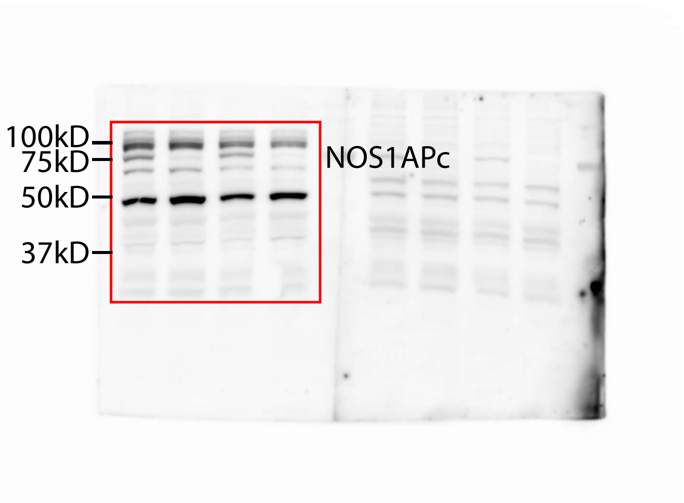

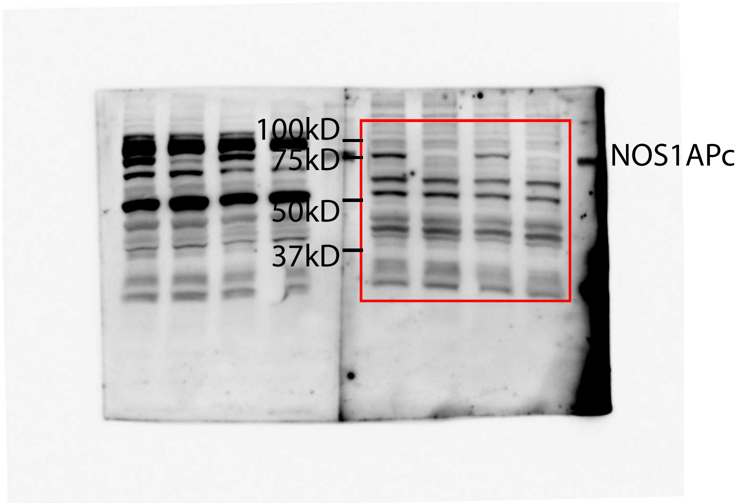

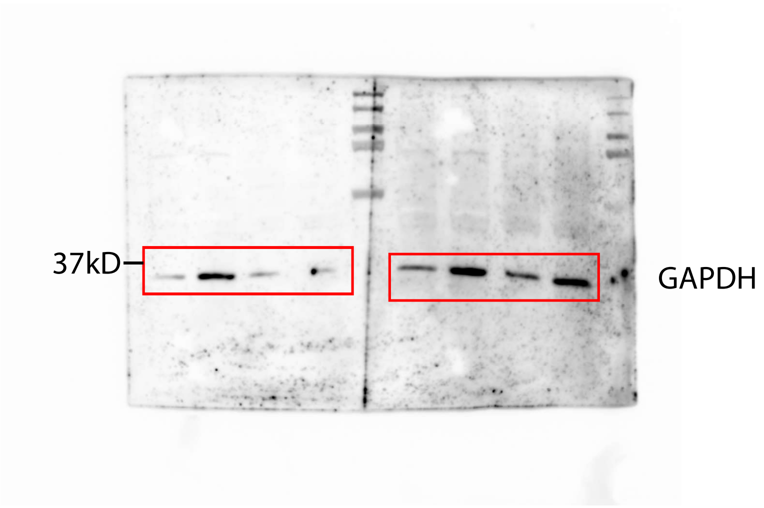

gel format: home-made, 10% SDS-PAGE

Supplementary Fig. 9a

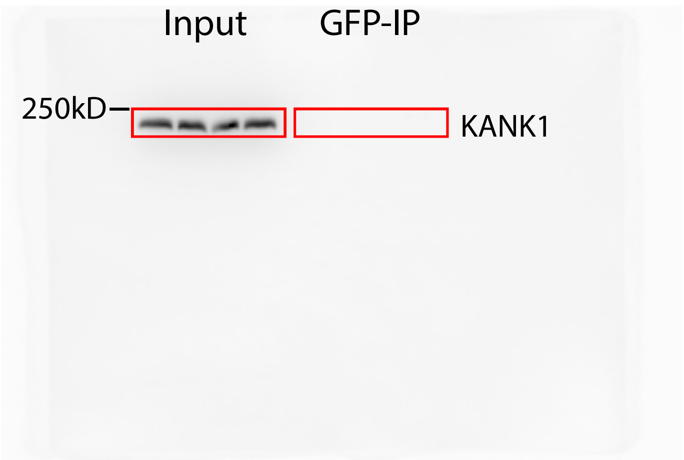

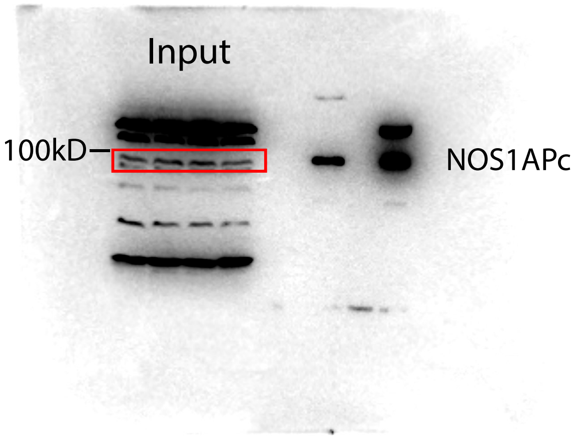

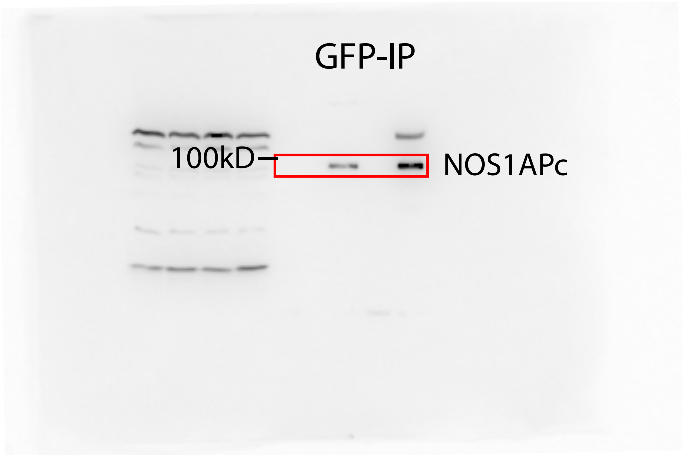

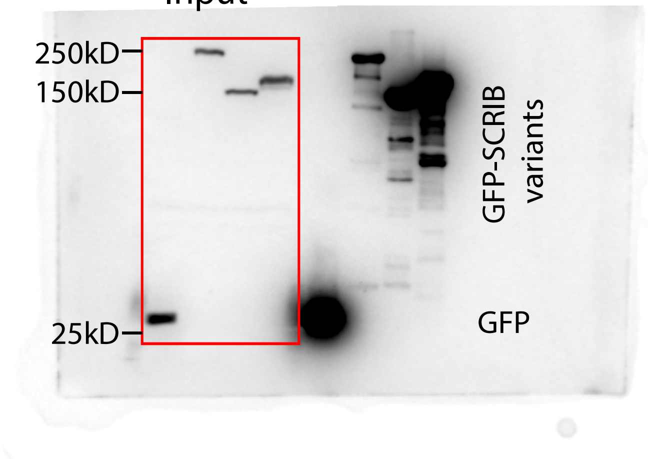

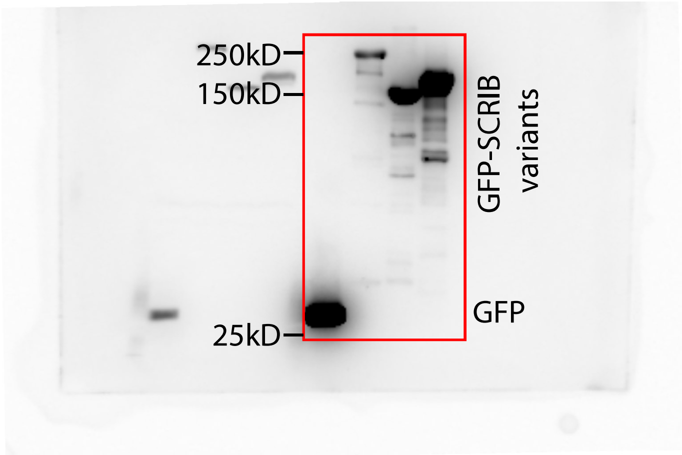

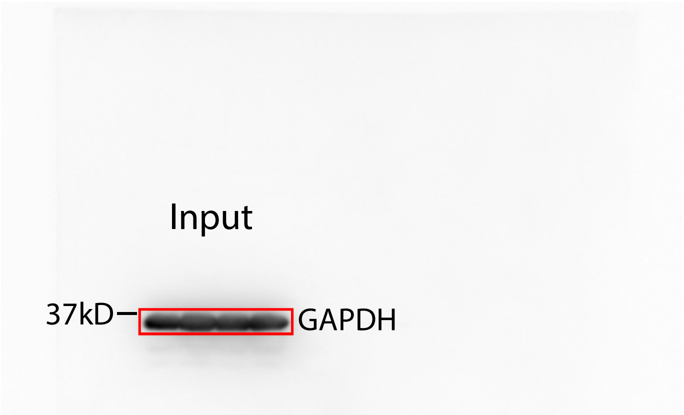

gel format: home-made, 10% SDS-PAGE

Supplementary Fig. 10b

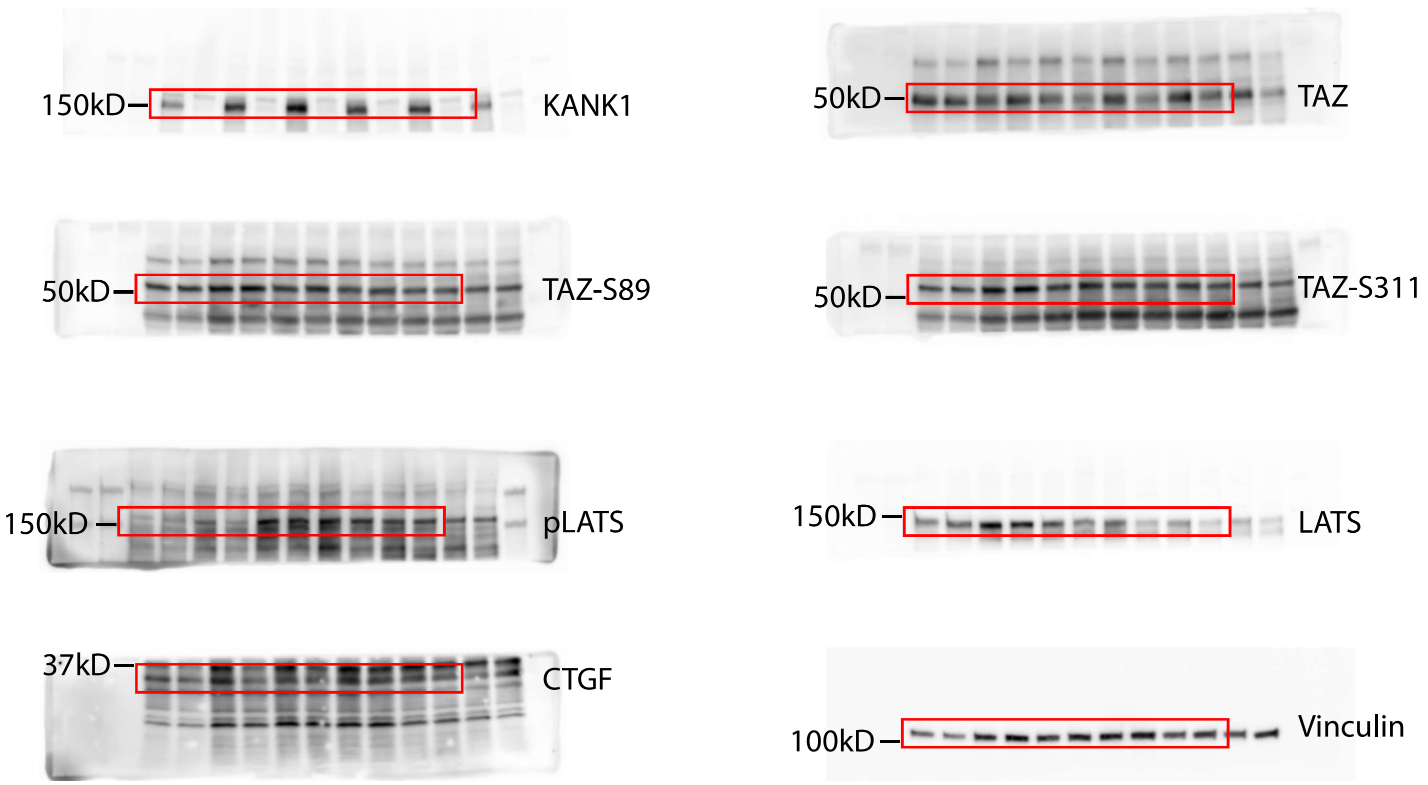

gel format: Bio-Rad (#4561086), gradient (4-15%) SDS-PAGE

Supplementary Fig. 11e

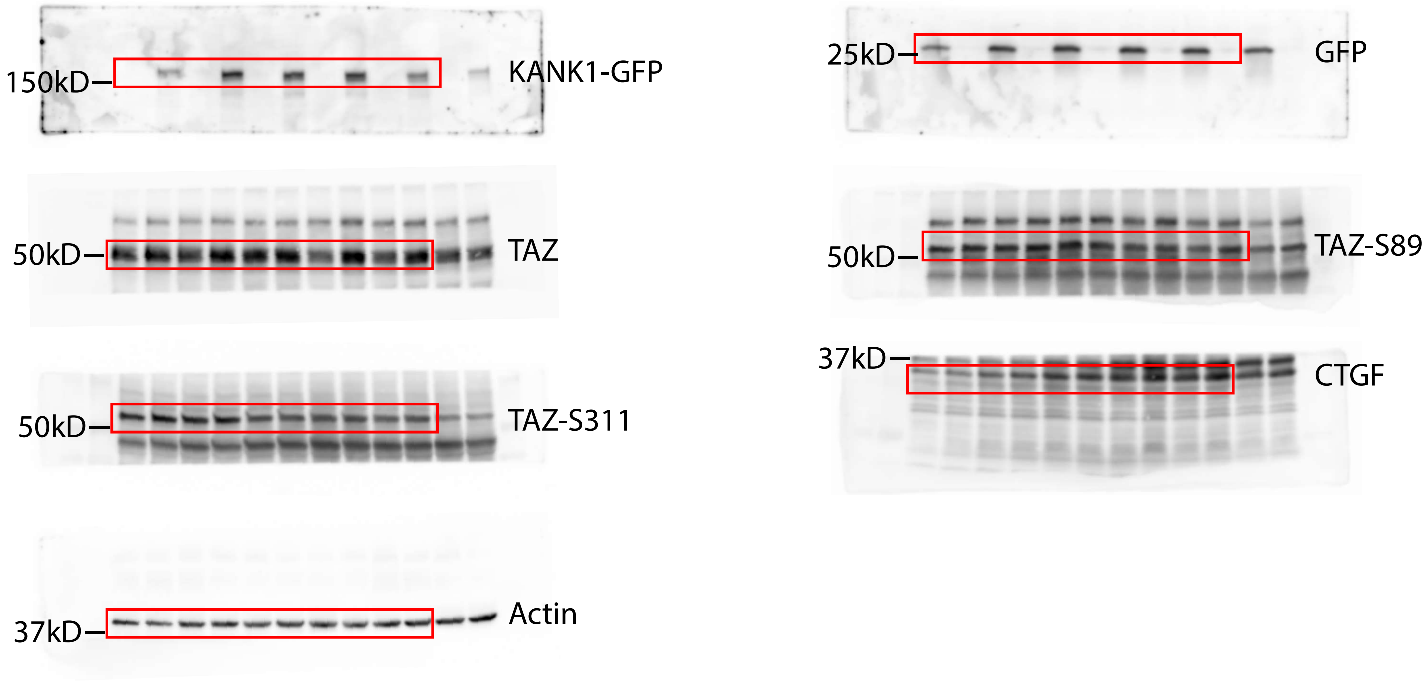

gel format: Bio-Rad (#4561086), gradient (4-15%) SDS-PAGE

Supplementary Fig. 12a

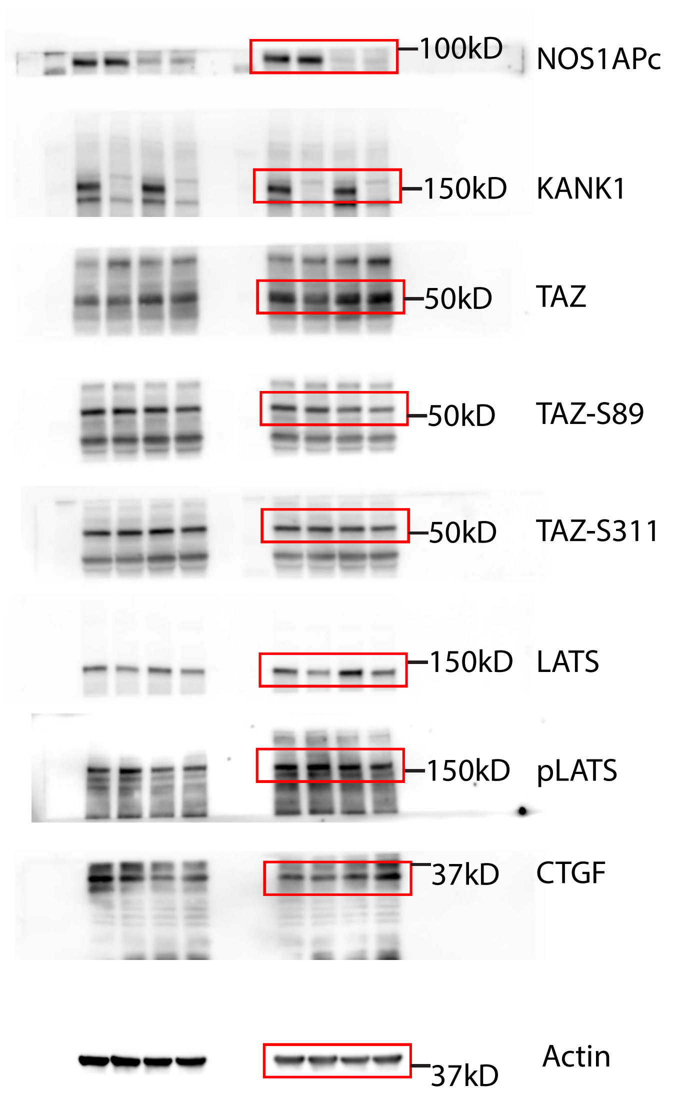

Supplementary Fig. 12c

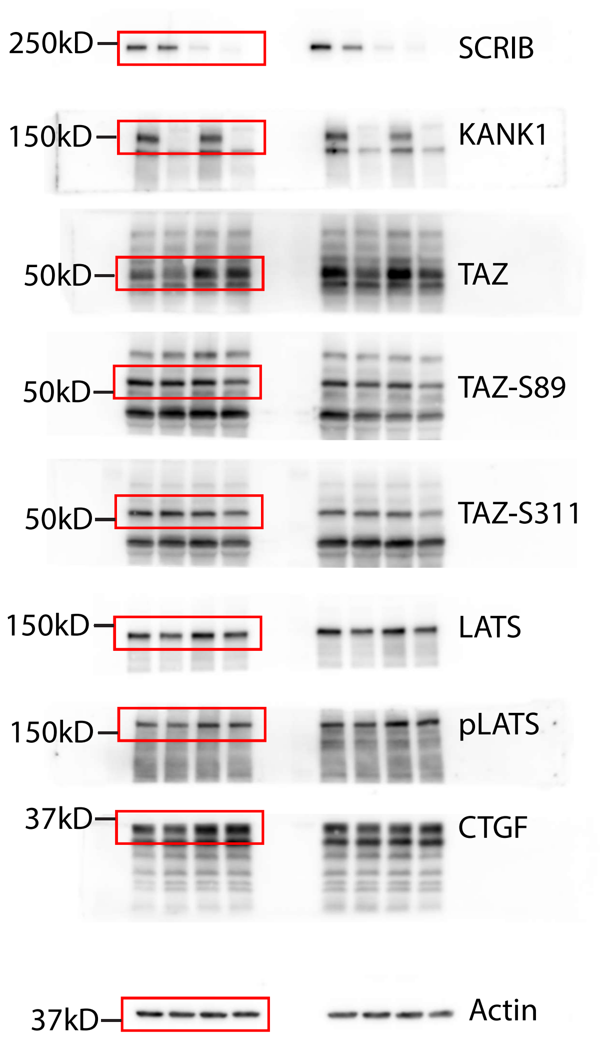

gel format: Bio-Rad (#4561086), gradient (4-15%) SDS-PAGE
